# Quantifying the Age of Evidence: Lessons From Cardiovascular Drugs

**DOI:** 10.1111/jep.70412

**Published:** 2026-03-19

**Authors:** Mariana B. Caiado Ferreira, Vinay Prasad

**Affiliations:** ^1^ Medicine 1, Unidade Local de Saúde São José Lisbon Portugal; ^2^ Department of Epidemiology and Biostatistics University of California San Francisco San Francisco CA USA

## Abstract

**Background:**

All evidence‐based medical decisions rely on the assumption that the evidence being used is applicable to current populations. To date, the age of evidence is not typically measured nor reported in systematic reviews.

**Methods:**

We sought to develop a method to quantify the age of evidence for medical interventions, using statins and sodium‐glucose co‐transporter protein 2 (SGLT‐2) inhibitors as exemplars, which may facilitate evaluating its potential outdatedness. We conducted a retrospective cross‐sectional study of all Cochrane reviews evaluating the effects of these drugs on cardiovascular disease (CVD) management up to August 2024, summarising the evidence and calculating the lag in time between patient enrolment in trials and current decision‐making (August 2024).

**Results:**

We identified 57 Cochrane reviews on statins, with 9 (15.8%) meeting our criteria, and 6 reviews on SGLT‐2 inhibitors, with 2 (33.3%) being eligible. The 9 statin reviews include 153 different trials, enrolling participants from 1988 to 2017, spanning 28 years. The 2 SGLT‐2 inhibitor reviews involve 67 trials from 2008 to 2021, spanning 13 years. The median age of evidence for statins is 24.1 years, compared to 8.6 years for SGLT‐2 inhibitors. Among the statin review topics, evidence was most robust for statins in the context of primary prevention of CVD.

**Conclusions:**

Our findings highlight the potential outdatedness of long‐established treatments like statins and suggest that reporting the age of evidence in systematic reviews may facilitate outdatedness assessments. We propose that future Cochrane reviews report the age of evidence, as this could significantly enhance the decision‐making process in evidence‐based medicine.

## 1 Introduction

All evidence‐based medical decisions rely on the assumption that the evidence being used is not outdated [1]. Thus, evidence generated at a given time needs to be applicable to patients living in the present. However, over time, patient characteristics and contexts change, which means that evidence becomes outdated. This explains why some therapies once supported by robust evidence are proven to no longer work. For instance, the declining efficacy of aspirin in the primary prevention of cardiovascular disease (CVD) may be partially attributed to participants in recent studies exhibiting healthier lifestyles and better management of risk factors and disease compared to those in trials from the 1980s [2].

Therefore, the age of evidence is a key factor to consider when making evidence‐based decisions. The age of evidence may serve, at least theoretically, as a proxy for the potential inapplicability of evidence due to changes in the patient population: the older the evidence, the less likely it is to apply in the present. However, the purpose of identifying the age of evidence goes beyond its implications for clinicians, the primary users of evidence. In fact, it may also extend to researchers, those involved in evidence synthesis, regulatory agencies and policymakers, all of whom can promote further research and periodic reassessments of evidence when evidence is deemed outdated.

While evidence‐based medicine is defined as the “explicit and judicious use of *current best* evidence” [1] (emphasis ours), it is possible that the “current best” research evidence is outdated when the population changes significantly over time. Time is certainly not the sole factor to take into consideration when assessing evidence “outdatedness”. For instance, by the time we wrote this paper in 2024, the evidence supporting the use of steroids in the treatment of severe COVID‐19 was only three to 4 years old [3]. Yet, this evidence was arguably outdated. COVID‐19 patients were already significantly different from the ones on whom studies were conducted. Most people infected with SARS‐CoV‐2 in 2024 had been vaccinated multiple times and/or have experienced prior infection, while study participants had neither. However, the accuracy of using the age of evidence as a proxy for outdatedness seemingly improves as time increases—the more time passes, the higher the likelihood that the evidence becomes outdated.

Currently, it is not standard practice in systematic reviews to calculate and report the age of evidence, despite its potential value. In the cardiovascular sphere, the age of the evidence supporting the use of statins in 2024 is unknown, despite statins being the cornerstone of pharmacological management of CVD since the 1990s [4]. However, the relevance of this evidence is currently questionable, given significant changes in the population's health profile, including decreased CVD death rates [5, 6], a dramatic drop in smoking [7], and rising rates of obesity and diabetes [5, 8]. Furthermore, advances in medical screening, diagnosis, and treatment have also altered the landscape of CVD management.

In this study, we sought to assess the age of evidence for statins and sodium‐glucose co‐transporter protein 2 (SGLT‐2) inhibitors in CVD management to demonstrate its value in assessing potential outdatedness. We examine all Cochrane reviews evaluating the effects of these drugs on CVD prevention, summarise the evidence and calculate the lag in time between patient enrolment and current decision‐making.

## 2 Methods

Taking cardiovascular disease prevention as an example, we sought to develop a method to quantify the timeliness of evidence for medical interventions by conducting a cross‐sectional analysis of Cochrane reviews for statins and SGLT‐2 inhibitors. As this was a descriptive study and not a systematic review of reviews or of their primary clinical trials, we have reported our study according to the Strengthening the Reporting of Observational Studies in Epidemiology (STROBE) reporting guideline for cross‐sectional studies. This study was not submitted for institutional review board approval because it did not involve health care records, and all data are publicly available.

### 2.1 Search Strategy and Selection Criteria

To identify all Cochrane reviews on the effects of statins or SGLT‐2 inhibitors in primary and/or secondary prevention of CVD in adults, the Cochrane Library was searched up to August 1, 2024. The search terms “statins” and “sodium‐glucose co‐transporter” were used, respectively. The Cochrane Library search interface returns only the latest, updated versions. As such, only the most recent versions of each review were included, thereby preventing duplication.

Reviews were included if they focused on statin/SGLT‐2 inhibitor therapy in adults or were broader in scope but included meta‐analyses for statins/SGLT‐2 inhibitors alone, and their primary outcomes included either cardiovascular mortality, cardiovascular events, or death from a specific major cardiovascular event (acute myocardial infarction, acute coronary syndrome, acute stroke or acute limb ischaemia). These criteria were chosen to ensure the selection of reviews with the most clinically relevant evidence of the effects of statins on CVD prevention. We excluded reviews on perioperative settings due to the short‐term nature of their outcomes, as well as those comparing different statin or SGLT‐2 inhibitor regimens.

One reviewer (M.C.F.) assessed the titles of all reviews identified and obtained the full‐text reviews for those potentially meeting the inclusion criteria and for which there was insufficient information in the title. Ambiguous cases were arbitrated with the other reviewer (V.P.).

### 2.2 Data Extraction and Analysis

From each included review, the first author (M.C.F.) extracted its date of publication, the total number of statin/SGLT‐2 inhibitor trials included, enrolment periods of included trials, sample size of each included trial, and effect size estimates for selected outcomes pertaining to comparisons of statins/SGLT‐2 inhibitors versus placebo, no treatment or usual care. The trials included in this study are listed in the Supplement (eTable 1 and eTable 2).

Summary effect size estimates were extracted for all‐cause mortality, cardiovascular mortality, total cardiovascular events, serious (non‐fatal) cardiovascular events, stroke events and coronary events outcomes, and statins' trial‐level estimates were extracted for the first three of these outcomes. Both summary and individual trial effect estimates (effect size and 95% confidence interval (CI)) were extracted from information presented in text, forest plots or tables in the review publication, with a preference for odds ratio (OR) or risk ratio (RR) metrics over hazard ratio (HR). The OR and RR are more often reported in Cochrane reviews, facilitating comparisons across studies and reviews. If neither were reported, but the HR was available, the HR was extracted. If trial‐level data were unavailable in the review, review authors were contacted. This occurred with two reviews (Aung et al, 2007: Lipid‐lowering for peripheral arterial disease of the lower limb; Taylor et al, 2013: “Statins for the primary prevention of cardiovascular disease”), but ultimately neither author provided the necessary data.

The date of publication of individual trials was extracted from the relevant study publication indicated in the references section in each review. If the exact date of publication was unextractable, the year of publication mentioned in the reference was used. Enrolment periods were always confirmed in trial‐related publications, when full texts were available. When enrolment periods were unspecified/unavailable (partly or completely), data were imputed. In such cases, enrolment start dates were assumed to be 2 years prior to the publication year, with completion assumed to occur 1 year before publication. Study identifications used in the reviews were generally preserved.

The age of evidence, that is, the time from evidence generation to the date of data abstraction (August 2024), was calculated for each trial by calculating the interval in years between the midpoint of the enrolment period and August 1, 2024. For each drug class (statins and SGLT‐2 inhibitors), the age of evidence was summarised using the median age of all included trials. The midpoint of the enrolment period for each trial was calculated by adding half the total duration of enrolment (in days) to the enrolment start date.

### 2.3 Statistical Analysis

Data extraction and analysis was done in Excel (Microsoft Corp) by a single investigator (M.C.F.). We conducted simple descriptive statistics to provide an overview of the features of the reviews and their included trials.

## 3 Results

**Figure 1:**
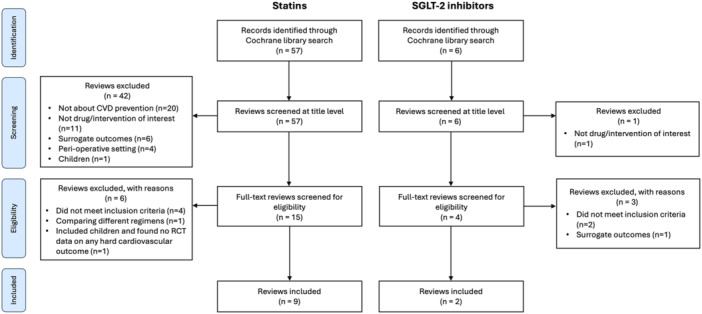
Flowchart of Cochrane review selection. CVD, cardiovascular disease; RCT, randomised controlled trial.

A total of 57 reviews from the Cochrane Library were identified for statins, of which 9 (15.8%) were included [9, 10, 11, 12, 13, 14, 15, 16, 17]. For SGLT‐2 inhibitors, the search yielded 6 results, of which 2 (33.3%) reviews were eligible for inclusion [18, 19]. A flowchart of review selection is shown in Figure 1. All reviews include only randomised controlled trials (RCTs) or quasi‐RCTs. Most reviews (*n* = 7, 63.6%) include RCTs only. Table 1 shows a list of all included reviews, their topics and the number of trials included in each review.

**Table 1:** General characteristics of Cochrane reviews included.

Year of publication	Topic	No. trials
**Statins**		
2007	Peripheral artery disease of the lower limb [9]	3*
2009	Prevention of stroke recurrence [11]	5*
2011	Acute ischaemic stroke [10]	8
2013	Primary prevention of cardiovascular disease [12]	18
2013	Chronic kidney disease requiring dialysis [13]	25
2014	Kidney transplant recipients [14]	22
2014	Acute coronary syndrome [15]	18
2016	Aortic valve stenosis [16]	4
2023	Chronic kidney disease not requiring dialysis [17]	63
**SGLT‐2 inhibitors**		
2021	Patients with cardiovascular disease [18]	13
2023	Chronic kidney disease and diabetes mellitus [19]	54**

* Number of trials in the review focusing on statins alone (the review included other lipid‐lowering therapies);

** Total number of studies included in the review, of which 36 were included in the quantitative analysis.

**Figure 2:**
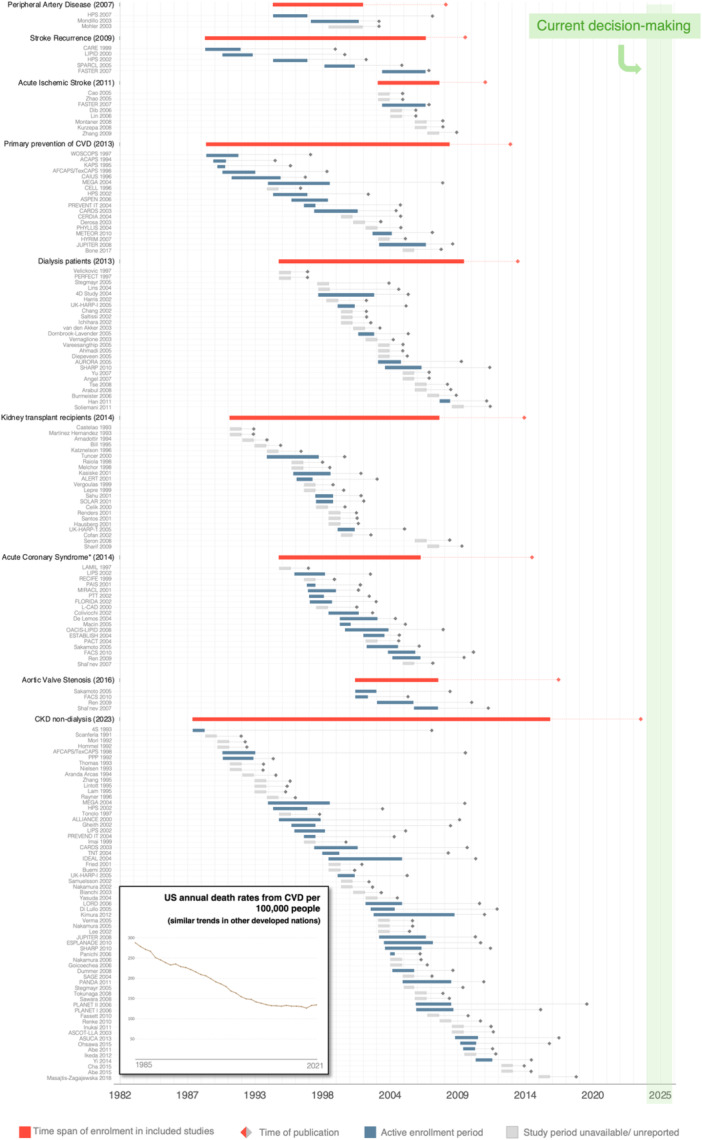
Time frame of statins' Cochrane reviews, enrolment periods and annual U.S. cardiovascular disease death rates (1985–2021). All statin trials included in the reviews are exhibited, regardless of the comparison group. Individual trials are presented by their enrolment start date in ascending order. The “time of publication” refers to the date when the Cochrane review or the individual trial was published, depicted as an orange or grey diamond, respectively. In the case of the individual trials, the “time of publication” is the date when the relevant analysis was first published (because some trials assessed different cardiovascular outcomes and thus were included in more than one review). The year in the study title corresponds to the year of publication of the relevant analysis included in the respective Cochrane review, and it may not correspond to the year of publication of the main trial analysis (some trials had secondary analyses reported at different time points). Annual death rates are age‐standardised, and similar downward trends are found in several other developed nations, as can be seen at the WHO Mortality Database (2025), in https://ourworldindata.org/grapher/cardiovascular-disease-death-rate-who-mdb?time=1985.2021&country=USA~GBR~DEU~PRT (accessed February 9, 2026). CVD, cardiovascular disease; U.S., United States.

The 9 statin reviews include a total of 153 different trials. Of the 153 trials, 10 (6.5%) were included in more than one review. The statin trials include patients enrolled between 1988 and 2017, spanning 29 years. Figure 2 illustrates the enrolment periods for individual trials included in the statin reviews, along with the overall enrolment period for each review (from the earliest enrolment start date to the latest enrolment completion date of the included trials). For additional context, the same figure also displays annual CVD death rates in the United States (U.S.) from 1985 to 2021, revealing a steady downward trend over the period. Study periods are unspecified or unavailable for 88 trials (56.4%).

**Figure 3:**
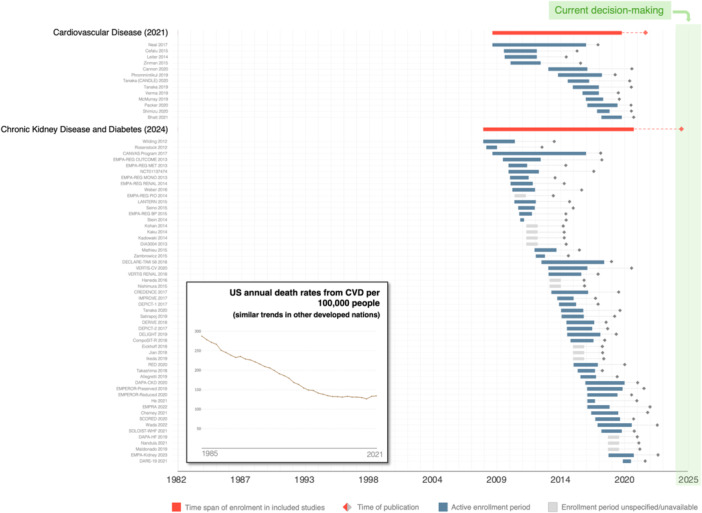
Time frame of SGLT‐2 inhibitors' Cochrane reviews, enrolment periods and annual U.S. cardiovascular disease death rates (1985‐2021). All SGLT‐2 inhibitor trials included in the reviews are exhibited, regardless of the comparison group. See Figure 2's legend. SGLT‐2, sodium‐glucose co‐transporter protein 2; U.S., United States.

The two SGLT‐2 inhibitor reviews include a total of 67 different trials. Patients in these trials were enrolled between 2008 and 2021, spanning 13 years. Figure 3 presents a time frame of SGLT‐2 inhibitor reviews and trials' enrolment periods akin to the one shown in Figure 2. Study periods are unspecified or unavailable for 13 trials (19.4%).

Table 2 shows the effect estimates for the selected outcomes assessed: all‐cause mortality, cardiovascular mortality death, total cardiovascular events, serious (non‐fatal) cardiovascular events, stroke events and coronary events. The table's colour coding indicates variability in the number of studies supporting each outcome estimate. Of the 7 statin reviews reporting all‐cause mortality and the 6 reviews reporting cardiovascular mortality, 2 reviews (28.6% and 33.3%, respectively) show statistically significant results for these outcomes, favouring statins. A reduction in total cardiovascular events is statistically significant in 3 statin reviews: “peripheral artery disease” review, “primary prevention of CVD” and “CKD not requiring dialysis”. As for SGLT‐2 inhibitors, both reviews show significant reductions in all‐cause mortality and cardiovascular mortality. Additionally, the 2021 review, which broadly covers CVD, further demonstrates significant reductions in total cardiovascular events and coronary events.

**Table 2:** Effect estimates for selected outcomes in statin and SGLT‐2 inhibitor Cochrane reviews.

					More Specific Outcomes		
Review Topic		All‐cause Mortality	CV Mortality	Total CV events	Serious (non‐fatal) CV events	Stroke events	Coronary events
(year of publication/no. of statin/SGLT‐2 inhibitor trials included)							
Statins	Peripheral Artery Disease [9] (2007; 3 trials)	—	—	**OR 0.74** a [0.67, 0.82]	—	—	**OR 0.77** a [0.66, 0.89]
	Stroke Recurrence [11] (2019; 5 trials)	OR 1.03^b^ [0.84, 1.25]	—	—	**OR 0.74** c [0.67, 0.82]	OR 0.88^d^ [0.77, 1.00]	—
	Acute Ischaemic Stroke [10] (2011; 8 trials)	OR 1.51^e^ [0.60, 3.81]	—	—	—	—	—
	Primary prevention of CVD [12] (2013; 18 trials)	**OR 0.86** g [0.79, 0.94]	**RR 0.83** d [0.72, 0.96]	**RR 0.75** h[0.70, 0.81]	—	**RR 0.78** i [0.68, 0.89]	**RR 0.73** j [0.67, 0.80]
	CKD requiring Dialysis [13] (2013; 25 studies)	RR 0.96^g^ [0.90, 1.02]	RR 0.94^g^ [0.84, 1.06]	RR 0.95^k^ [0.88, 1.03]	—	RR 1.29^a^ [0.96, 1.72]	RR 0.87^c^^,^ ** [0.71, 1.07]
	Kidney Transplant Recipients [14] (2014; 22 studies)	RR 1.08^f^ [0.85, 1.63]	RR 0.68^k^ [0.45, 1.01]	RR 0.84^b^ [0.66, 1.06]	—	RR 1.18^b^ [0.85, 1.63]	—
	Acute Coronary Syndrome* [15] (2014) (total of 18 studies)	OR 0.90^l^ [0.70, 1.14]	RR 0.84^m^ [0.64, 1.09]	OR 0.93^n^ [0.81, 1.06]	—	RR 0.72^e^ [0.45, 1.16]	RR 0.91^I^^,^ ** [0.77, 1.06]
	Aortic Valve Stenosis [16] (2016; 4 studies)	—	RR 0.80^c^ [0.56, 1.15]	—	—	—	—
	CKD not requiring Dialysis [17] (2023; 63 studies)	**RR 0.83** g [0.73, 0.96]	**RR 0.77** m [0.69, 0.87]	**RR 0.72** j [0.66, 0.79]	—	RR 0.64^e^ [0.37, 1.08]	**RR 0.55** I ^,^ ** [0.42, 0.73]
SGLT‐2 inhibitor	Cardiovascular Disease [18] (20021; 31 studies)	**OR 0.84** d [0.74, 0.96]	**OR 0.82** d [0.70, 0.95]	**OR 0.82** k [0.70, 0.96]	—	OR 1.12^d^ [0.92, 1.36]	**OR 0.97** a ^,^ * [0.84, 0.96]
	CKD and Diabetes [19] (2024; 53 studies)	**RR 0.85** ° [0.78, 0.94]	**RR 0.83** g [0.74, 0.93]	—	—	OR 1.07^a^ [0.88, 1.30]	RR 0.95^a^^,^ * [0.80, 1.14]

*Note:* The effect estimates pertain to comparisons of statins versus placebo, no treatment or usual care. Effect measures bolded and underlined are statistically significant.

Abbreviations: CKD, chronic kidney disease; CVD, cardiovascular disease; OR, odds ratio; RR, risk ratio.

* Short‐term outcomes (3 to 6 months).

** Coronary outcome: myocardial infarction (fatal or non‐fatal).

^a^ 2 studies;

^b^ 1 study;

^c^ 3 studies;

^d^ 5 studies;

^e^ 7 studies;

^f^ 6 studies;

^g^ 13 studies;

^h^ 9 studies;

^i^ 10 studies;

^j^ 14 studies;

^k^ 4 studies;

^l^ 12 studies;

^m^ 8 studies;

^n^ 11 studies,

^°^ 20 studies. The colour shading is based on the number of studies included: five shades of blue, ranging from lightest (1–3 studies) to darkest (19–21 studies), increasing in tone every three studies.

**Figure 4:**
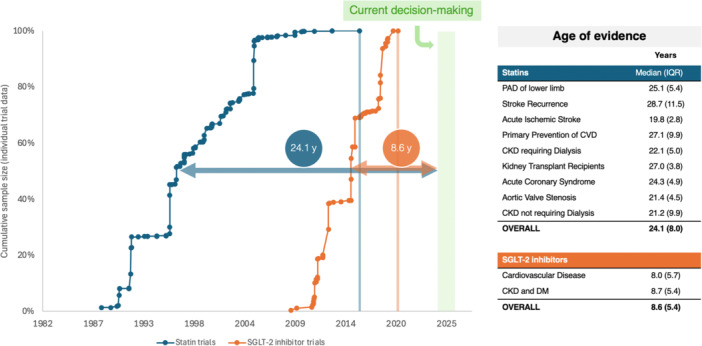
Comparison of statins and SGLT‐2 inhibitors' age of evidence. Each point estimate corresponds to the midpoint of the enrolment period for an individual trial. CKD, chronic kidney disease; CVD, cardiovascular disease; DM, diabetes mellitus; IQR, interquartile range; PAD, peripheral artery disease; SGLT‐2, sodium‐glucose co‐transporter protein 2.

For statins, the median overall time from evidence generation to present time is 24.1 (IQR 8.0) years, whereas for SGLT‐2 inhibitors it is 8.6 (IQR 5.4) years (Figure 4). Among the statin review topics, acute ischaemic stroke bears the most recent evidence (19.8 years) and stroke recurrence the oldest (28.7 years).

## 4 Discussion

To the best of our knowledge, ours is the first attempt to quantify the age of medical evidence. Our method seeks to capture the lag time between evidence generation, represented by the midpoint in the enrolment period, and current decision‐making. By providing an objective indication of the age of evidence, one can more effectively evaluate the potential outdatedness of that evidence. We also developed a graphical method to convey this to readers (Figures 2, 3, 4).

As of August 2024, nine and two Cochrane reviews offer guidance on the use of statins and SGLT‐2 inhibitors, respectively, for adult CVD management. We found that the body of evidence supporting statin therapy in nine different contexts is 24 years old. In contrast, the body of evidence supporting SGLT‐2 inhibitors is only 9 years old. Notably, the most recent enrolment period among statin trials was completed around 2017, whereas for SGLT‐2 inhibitors it was in 2021.

While statins have proved to have robust effects in hard cardiovascular outcomes across a variety of patient contexts, particularly in primary prevention, our findings raise important questions about the relevance of this evidence for current clinical practice. The patient population has changed considerably over the last few decades. The fact that cardiovascular death rates declined considerably from 1985 to 2021 in the U.S. (as shown in Figure 2) and other developed nations likely reflects those changes. Moreover, since most statin trials were conducted, risk factor management has improved (e.g., blood pressure [5]) and newer drugs have become part of the cardiovascular therapeutic armamentarium. This includes not only SGLT‐2 inhibitors, whose benefits we have demonstrated, but also glucagon‐like peptide‐1 (GLP‐1) agonists [18]. In the U.S., from 1990 to 2019, age‐standardised rates of “years of life lived with disability or injury” attributable to high body mass index and high fasting plasma glucose increased by 44.4% and 47.4%, respectively, making them the leading risk factors [5]. In Europe, overweight and obesity have also become the leading behavioural risk factors for disability [20].

Given these shifts in population health and advancements in treatment, the fact that the age of evidence supporting statin use is 24 years old suggests that this evidence may actually be outdated. In contrast, the more recent evidence supporting SGLT‐2 inhibitors is far more likely to apply to present‐day patients.

Our findings are particularly relevant also because statins are among the most commonly prescribed drugs. Between 2019 and 2021, atorvastatin was the most prescribed drug in the United Kingdom and the second most in the U.S. between 2017 and 2019 [21]. This emphasises the importance of periodically revisiting the evidence behind established practices. Evidence‐based medicine is best practised if clinical decisions are based on relevant and up‐to‐date evidence. Routinely reporting the age of evidence in systematic reviews could also assist guidelines authors in the evaluation of the evidence base for a given intervention. A clear and transparent comment on the age of evidence, particularly if made in a proper guideline section, may better inform guidelines users.

This study has limitations. First, no reviews were found addressing chronic coronary syndromes, which are also a major area of statin therapy use. While Cochrane is considered a very reliable and often updated source of high‐quality evidence, it is possible that other important areas of use of statins were not covered and relevant trials were missed. Second, there was a significant amount of missing data regarding statin enrolment periods (57%) that required data imputation. However, we do not anticipate this having a major impact on our findings, as we imputed conservative estimates. Alternatively, enrolment could only have occurred before the imputed dates, which would only extend the lag between evidence generation and the present time. Third, there is likely some heterogeneity in study populations and outcome definitions between the reviews, which may somewhat limit comparisons between outcome estimates.

## 5 Conclusion

We develop a method to quantify the age of evidence for a current health practice from prior randomised trials, using the case of statins and SGLT‐2 inhibitors. We presented a simple method of measuring and visually presenting the age of evidence, which may be readily implemented in Cochrane systematic reviews, as it does not require any additional data collection. We propose that all future Cochrane reviews report the age of evidence not only numerically but also visually, using timelines similar to those we presented. We believe these tools can be highly valuable not only for health practitioners but also for researchers and policymakers committed to evidence‐based medicine.

## Author Contributions

**Mariana B. Caiado Ferreira:** concept and design, acquisition, analysis, interpretation of data, drafting of the manuscript, critical review of the manuscript for important intellectual content and statistical analysis. **Vinay Prasad:** concept and design, critical review of the manuscript for important intellectual content and supervision.

## Conflicts of Interest

This article was prepared when Dr. Prasad was an employee of the University of California, San Francisco, prior to his employment at the U.S. Food and Drug Administration (FDA), and does not represent the views of the FDA. At the time, V.P. received research funding from Arnold Ventures through a grant made to UCSF, and royalties for books and writing from Johns Hopkins Press, MedPage, and the Free Press. V.P. declared consultancy roles with UnitedHealthcare and OptumRX; V.P hosted the podcasts, Plenary Session, VPZD, Sensible Medicine, wrote the newsletters, Sensible Medicine, the Drug Development Letter and VP's Observations and Thoughts, and ran the YouTube channel Vinay Prasad MD MPH, which collectively earn revenue on the platforms: Patreon, YouTube and Substack.

## Supporting information


**eTable 1:** List of studies in each Cochrane Statins Review. **eTable 2:** List of studies in each Cochrane SGLT‐2 inhibitor Review.

## Data Availability

The data that support the findings of this study are available from the corresponding author upon reasonable request.
