# Supplementary material for: Quantifying the Age of Evidence: Lessons From Cardiovascular Drugs
Source: J Eval Clin Pract. 2026 Mar 19;32(2):e70412. doi: 10.1111/jep.70412 (PMC13002139; doi:10.1111/jep.70412)
Supplement: Supplementary file 1 — eTable 1: List of studies in each Cochrane Statins Review. eTable 2: List of studies in each Cochrane SGLT‐2 inhibitor Review. [file JEP-32-0-s001.docx]

**Supplement**

[eTable 1. List of studies in each Cochrane Statins Review 2](#_Toc221556114)

[eTable 2. List of studies in each Cochrane SGLT-2 inhibitor Review 15](#_Toc221556115)

| eTable 1. List of studies in each Cochrane Statins Review | | |
| --- | --- | --- |
|  | **Trial** | **Reference** |
| **Peripheral artery disease of the lower limb (2007)** | | |
|  | HPS 2002 | Heart Protection Study Collaborative Group. MRC/ BHF Heart Protection Study of cholesterol lowering with simvastatin in 20,536 high-risk individuals: a randomised placebo-controlled trial. *Lancet* 2002;**360** (9326):7–22. |
|  | Mohler 2003 | Mohler ER 3rd, Hiatt WR, Creager MA. Cholesterol reduction with atorvastatin improves walking distance in patients with peripheral arterial disease. *Circulation* 2003; **108**(12):1481–6. |
|  | Mondillo 2003 | Mondillo S, Ballo P, Barbati R, Guerrini F, Ammaturo T, Agricola E, et al.Effects of simvastatin on walking performance and symptoms of intermittent claudication in hypercholesterolemic patients with peripheral vascular disease. *American Journal of Medicine* 2003;**114**(5):359–64. |
| **Prevention of stroke recurrence (2009)** | | |
|  | CARE 1999 | Plehn JF, Davis BR, Sacks FM, Rouleau JL, Pfeffer MA, Bernstein V, et al. Reduction of stroke incidence after myocardial infarction with pravastatin: the Cholesterol and Recurrent Events (CARE) Study. *Circulation* 1999;**99**(2): 216–23. [MEDLINE: 99110724] |
|  | FASTER 2007 | Kennedy J, Hill MD, Ryckborst KJ, Eliasziw M, Demchuk AM, Buchan AM. Fast assessment of stroke and transient ischaemic attack to prevent early recurrence (FASTER): a randomised controlled pilot trial. *Lancet Neurology* 2007;**6**: 961–9. |
|  | HPS 2002 | Heart Protection Study Collaborative Group. Effects of cholesterol-lowering with simvastatin on stroke and other major vascular events in 20,536 people with cerebrovascular disease or other high-risk conditions. *Lancet* 2004;**363**: 757–67. |
|  | LIPID 2000 | White HD, Simes J, Anderson NE, Hankey GJ, Watson JDG, Hunt D, et al. Pravastatin therapy and the risk of stroke. *New England Journal of Medicine* 2000;**343**(5): 317–26. |
|  | SPARCL 2005 | SPARCL Investigators. High-dose atorvastatin after stroke or transient ischemic attack. *New England Journal of Medicine* 2006;**355**(6):549–59. [MEDLINE: 16899775] |
| **Acute ischemic stroke (2011)** | | |
|  | Cao 2005 | Cao H, Sun CK, Zhao GY, Tang SL, Wang QY, Hou Y, et al.Effect of statins on neurological impairment and correlative parameters in serum in patients with cerebral infarction. *Chinese Journal of Clinical Rehabilitation* 2005; **9**:94–6 |
|  | Dib 2006 | Dib H, Horozoglu H, Afsar N, Ekinci G, Aktan S. The effect of statins on infarct size and prognosis. *Cerebrovascular Diseases* 2006;**21 Suppl 4**:146. |
|  | FASTER 2007 | Kennedy J, Hill MD, Eliasziw M, Demchuk AW, Buchan AM, FASTER Investigators. Fast assessment of stroke and transient ischaemic attack to prevent early recurrence (FASTER): a randomised controlled pilot trial. *Lancet Neurology* 2007;**6**(11):961–9. |
|  | Kurzepa 2008 | Kurzepa J, Bielewicz J, Bartosik-Psujek H, Szczepanska- Szerej A, Stelmasiak Z. Simvastatin inhibits the increase in serum tau protein levels in the acute phase of ischemic stroke. *Pharmacological Reports* 2008;**60**(6):1014–8. |
|  | Lin 2006 | Lin YZ, Sun CK, Xiao ZY, Song GR, Yin L, Bao LP. Effects of atorvastatin in different courses of treatment on blood fat and neurologic impairment in patients with cerebral infarction. *Chinese Journal of Clinical Rehabilitation* 2006; **10**:28–31. |
|  | Montaner 2008 | Montaner J, Chacon P, Krupinski J, Rubio F, Millan M, Molina CA, et al.Simvastatin in the acute phase of ischemic stroke: a safety and efficacy pilot trial. *European Journal of Neurology* 2008;**15**(1):82–90. |
|  | Zhang 2009 | Zhang J. Effects of different doses of atorvastatin on lipid levels and nervous function in patients with acute cerebral infarction. *Chinese Journal of Contemporary Neurology and Neurosurgery* 2009;**9**:46–9. |
|  | Zhao 2005 | hao SP, Wu J, Tan LM, Hu ZP, Xiao ZJ, Nie S, et al.Effects of atorvastatin on plasma hypersensitive C-reactive protein and interleukin-6 in patients with acute cerebral infarction. *National Medical Journal of China* 2005;**85**(40):2841–5. |
| **Primary prevention of cardiovascular disease (2013)** | | |
|  | ACAPS 1994 | Furberg C, Adams HP Jr, Appelgate WB, Byington RP, Espeland MA, Hartwell T, et al.Effect of lovastatin on early carotid atherosclerosis and cardiovascular events. Asymptomatic Carotid Artery Progression Study (ACAPS) Research Group. *Circulation* 1994;**90**(4):1679–87. |
|  | MEGA 2004 | Brookes L, Nakamura H. MEGA: Management of elevated cholesterol in the primary prevention group of adult Japanese. American Heart Association 2005 Scientific Sessions 2008 |
|  | AFCAPS/TexCAPS 1998 | Downs JR, Clearfield M, Weis S, Whitney E, Shapiro DR, Beere PA, et al.Primary prevention of acute coronary events with lovastatin in men and women with average cholesterol levels: results of AFCAPS/TexCAPS. Air Force/ Texas Coronary Atherosclerosis Prevention Study. *JAMA* 1998;**279**(20):1615–22. |
|  | ASPEN 2006 | Knopp RH, D’Emden M, Smilde JG, Pocock SJ, on behalf of the ASPEN Study Group. Efficacy and safety of atorvastatin in the prevention of cardiovascular end points in subjects with type 2 diabetes: the Atorvastatin Study for Prevention of Coronary Heart Disease Endpoints in non-insulin-dependent diabetes mellitus (ASPEN). *Diabetes Care* 2006;**29**(7):1478–85. |
|  | Bone 2007 | Bone HG, Kiel DP, Lindsay RS, Lewiecki EM, Bolognese MA, Leary ET, et al.Effects of atorvastatin on bone in postmenopausal women with dyslipidemia: A double-blind, placebo-controlled, dose-ranging trial. *Journal of Clinical Endocrinology and Metabolism* 2007;**92**(12):4671–77. |
|  | CAIUS 1996 | Mercuri M, Bond G, Sirtori CR, Veglia F, Crepaldi  G, Feruglio S, et al.Pravastatin reduces carotid intima- media thickness progression in an asymptomatic hypercholesterolemic Mediterranean population: the Carotid Atherosclerosis Italian Ultrasound Study. *American Journal of Medicine* 1996;**101**:627–34. |
|  | CARDS 2003 | Colhoun HM, Betteridge DJ, Durrington PN, Hitman GA, Neil HAW, Livingstone SJ, et al.Primary prevention of cardiovascular disease with atorvastatin in type 2 diabetes in the Collaborative Atorvastatin Diabetes Study (CARDS): multicentre randomised placebo-controlled trial. *Lancet* 2004;**364**:685–96. |
|  | CELL 1996 | Lindholm LH, Ekbom T, Dash C, Isacsson A, Schersten B, for the CELL Study Group. Changes in cardiovascular risk factors by combined pharmacological strategies: the main results of the CELL Study. *Journal of Internal Medicine* 1996;**240**:13–22. |
|  | CERDIA 2004 | Beishuizen ED, Van De Ree MA, Jukema JW, Tamsma JT, van der Vijver JC, Meinders AE, et al.Two year statin therapy does not alter the progression of intima medica thickness in patients with type 2 diabetes without manifest cardiovascular disease. *Diabetes Care* 2004;**27**(12):2887–91. |
|  | Derosa 2003 | Derosa G, Mugellini A, Ciccarelli L, Fogari R. Randomized, double-blind, placebo-controlled comparison of the action of orlistat, fluvastatin, or both on anthropometric measurements, blood pressure, and lipid profile in obese patients with hypercholesterolemia prescribed a standardized diet. *Clinical Therapeutics* 2003;**25**(4):1107–22. |
|  | HYRIM 2007 | Sigmund A, Hjelstuen AK, Hjermann I, Bjerkan K, Holme I. Fluvastatin and lifestyle modification for reduction of carotid intima-media thickness and left ventricular mass progression in drug-treated hypertensives. *Atherosclerosis* 2004;**178**:387–97. |
|  | JUPITER 2008 | Ridker PM, Danielson E, Fonseca FAH, Genest J, Gotto AM Jr, Kastelein JJ, et al.JUPITER Study Group. Rosuvastatin to prevent vascular events in men and women with elevated C-Reactive protein. *New England Journal of Medicine* 2008;**359**(21):2195–207. |
|  | KAPS 1995 | Salonen R, Nyyssonen K, Porkkala E, Rummukainen J, Belder R, Park J-S, et al.Kuopio Atherosclerosis Prevention Study (KAPS). A population-based primary preventive trial of the effect of LDL lowering on atherosclerotic progression in carotid and femoral arteries. *Circulation* 1995;**92**: 1758–64. |
|  | METEOR 2010 | Crouse JR 3rd, Raichlen JS, Riley WA, Evans GW, Palmer MK, O’Leary DH, et al.METEOR Study Group. Effect of rosuvastatin on progression of carotid intima- media thickness in low-risk individuals with subclinical atherosclerosis: The METEOR Trial. *JAMA* 2007;**297**(12): 1344–53. |
|  | HPS 2002 | Heart Protection Study Collaborative Group. Randomised trial of the effects of cholesterol-lowering with simvastatin on peripheral vascular and other major vascular outcomes. *Journal of Vascular Surgery* 2007;**45**:645–54. |
|  | PHYLLIS 2004 | Zanchetti A, Crepaldi G, Bond M, Gallus G, Veglia F, Mancia G, et al.PHYLLIS Investigators. Different effects of anti-hypertensive regimens based on fosinopril or hydrochlorothiazide with or without lipid lowering pravastatin on progression of asymptomatic carotid atherosclerosis: principal results of PHYLLIS- a randomised double blind trial. *Stroke* 2004;**35**(12):2807–12. |
|  | PREVEND IT 2004 | Asselbergs FW, Diercks GFH, Hillege HL, van Boven AJ, Janssen WMT, Voors AA, et al.Effects of fosinopril and pravastatin on cardiovascular events in subjects with microalbinuria. *Circulation* 2004;**110**:2809–16. Asselbergs FW, Hillege HL, Van Gilst WH. Framingham score and microalbuminuria: combined future targets for primary prevention?. *Kidney international.Supplement* 2004; **66**(supplement 92):S111–4. |
|  | WOSCOPS 1997 | The West of Scotland Coronary Prevention Study Group. Baseline risk factors and their associations with outcome in the West of Scotland Coronary Prevention Study. *American Journal of Cardiology* 1997;**79**:756–62. |
| **Chronic kidney disease requiring dialysis (2013)** | | |
|  | 4D Study | Wanner C, Krane V, März W, Olschewski M, Mann JF, Ruf G, et al. Atorvastatin in patients with type 2 diabetes mellitus undergoing hemodialysis. *New England Journal of Medicine* 2005;**353**(3):238-48. [MEDLINE: 16034009] |
|  | Ahmadi 2005 | Ahmadi FL, Eslami K, Maziar S, Lessan-Pezeshki M, Reza- Khatami M, Mahdavi-mazdeh M, et al. Effect of lovastatin on C-reactive protein and hemoglobin in hemodialysis patients [abstract]. *Nephrology* 2005;**10**(Suppl):A283-4. |
|  | Angel 2007 | Angel JR, Rojas E, del Campo FM, Nava D, Cueto-Manzano A. Effect of pravastatin on inflammatory status of CAPD patients: a randomized, double-blinded, controlled and cross-over clinical trial [abstract]. *Journal of the American Society of Nephrology* 2007;**18**(Abstracts):270A |
|  | Arabul 2008 | Arabul M, Gullulu M, Yilmaz Y, Akdag I, Kahvecioglu S, Eren MA, et al. Effect of fluvastatin on serum prohepcidin levels in patients with end-stage renal disease. *Clinical Biochemistry* 2008;**41**(13):1055-8. [MEDLINE: 18571502] |
|  | AURORA 2005 | Fellstrom BC, Jardine AG, Schmieder RE, Holdaas H, Bannister K, Beutler J, et al. Rosuvastatin and cardiovascular events in patients undergoing hemodialysis. *New England Journal of Medicine* 2009;**360**(14):1395-407. [MEDLINE: 19332456] |
|  | Burmeister 2006 | Burmeister JE, Miltersteiner DR, Campos BM. Rosuvastatin in hemodialysis: short-term effects on lipids and C-reactive protein. *Journal of Nephrology* 2009;**22**(1):83-89. [MEDLINE: 19229822] |
|  | Chang 2002 | Chang JW, Yang WS, Min WK, Lee SK, Park JS, Kim SB. Effects of simvastatin on high-sensitivity C-reactive protein and serum albumin in hemodialysis patients. *American Journal of Kidney Diseases* 2002;**39**(6):1213-7. [MEDLINE: 12046033] |
|  | Diepeveen 2005 | Diepeveen SH, Verhoeven GH, van der Palen J, Dikkeschei LD, Denmacker PN, Kolsters G, et al. Effects of atorvastatin and vitamin E on lipoproteins and oxidative stress in dialysis patients: a randomised-controlled trial. *Journal of Internal Medicine* 2005;**257**(5):438-45. [MEDLINE: 15836660] |
|  | Dornbrook-Lavender 2005 | Dornbrook-Lavender KA, Joy MS, Denu-Ciocca CJ, Chin H, Hogan SL, Pieper JA. Effects of atorvastatin on low-density lipoprotein cholesterol phenotype and C-reactive protein levels in patients undergoing long-term dialysis. *Pharmacotherapy* 2005;**25**(3):335-44. [MEDLINE: 15843280] |
|  | Han 2011 | Han SH, Kang EW, Yoon SJ, Lee HC, Yoo TH, Choi KH, et al. Combined vascular effects of HMG-CoA reductase inhibitor and angiotensin receptor blocker in non-diabetic patients undergoing peritoneal dialysis. *Nephrology Dialysis Transplantation* 2011;**26**(11):3722-8. [MEDLINE: 21385862] |
|  | Harris 2002 | Harris KP, Wheeler DC, Chong CC, Atorvastatin in CAPD Study Investigators. A placebo controlled trial examining atorvastatin in dyslipidemic patients undergoing CAPD. *Kidney International* 2002;**61**(4):1469-74. [MEDLINE: 11918754] |
|  | Ichihara 2002 | Ichihara A, Hayashi M, Ryuzaki M, Handa M, Furukawa T, Saruta T. Fluvastatin prevents development of arterial stiffness in haemodialysis patients with type 2 diabetes mellitus. *Nephrology Dialysis Transplantation* 2002;**17**(8):1513-7. [MEDLINE: 12147804] |
|  | Lins 2004 | Lins RL, Matthys KE, Billiouw JM, Dratwa M, Dupont P, Lameire NH, et al. Lipid and apoprotein changes during atorvastatin up-titration in hemodialysis patients with hypercholesterolemia: a placebo-controlled study. *Clinical Nephrology* 2004;**62**(4):287-94. [MEDLINE: 15524059] |
|  | PERFECT 1997 | Walker RJ, Sutherland WH, Walker HL, MacMahon S, Robson RA. Effect of treatment with simvastatin on serum cholesteryl ester transfer in patients on dialysis. PERFECT Study Collaborative Group. *Nephrology Dialysis Transplantation* 1997;**12**(1):87-92. [MEDLINE: 9027779] |
|  | Saltissi 2002 | altissi D, Morgan C, Rigby RJ, Westhuyzen J. Safety and efficacy of simvastatin in hypercholesterolemic patients undergoing chronic renal dialysis. *American Journal of Kidney Diseases* 2002;**39**(2):283-90. [MEDLINE: 11840368] |
|  | SHARP 2010 | Baigent C, Reith C, Emberson J, Wheeler DC, Tomson CR, Wanner C, et al. The effects of lowering LDL cholesterol with simvastatin plus ezetimibe in patients with chronic kidney disease (Study of Heart and Renal Protection): a randomised placebo-controlled trial. *Lancet* 2011;**377**(9784):2181-92. [MEDLINE: 21663949] |
|  | Soliemani 2011 | Soliemani A, Nikoueinejad H, Tabatabaizade M, Mianehsaz E, Tamadon M. Effect of hydroxymethylglutaryl-CoA reductase inhibitors on low-density lipoprotein cholesterol, interleukin-6, and high-sensitivity C-reactive protein in end-stage renal disease. *Iranian journal of Kidney Diseases* 2011;**5**(1):29-33. [MEDLINE: 21189431] |
|  | Stegmayr 2005 | Stegmayr BG, Brännström M, Bucht S, Crougneau V, Dimeny E, Ekspong A, et al. Low-dose atorvastatin in severe chronic kidney disease patients: a randomized, controlled endpoint study. *Scandinavian Journal of Urology & Nephrology* 2005;**39**(6):489-97. [MEDLINE: 18161210] |
|  | Tse 2008 | Tse KC, Yung S, Tang CS, Tam S, Lai KN, Chan TM. Atorvastatin at conventional dose did not reduce C-reactive protein in patients on peritoneal dialysis. *Journal of Nephrology* 2008;**21**(3):283. [MEDLINE: 18587714] |
|  | UK-HARP-I 2005 | Baigent C, Landray M, Leaper C, Altmann P, Armitage J, Baxter A, et al. First United Kingdom Heart and Renal Protection (UK-HARP-I) study: biochemical efficacy and safety of simvastatin and safety of low-dose aspirin in chronic kidney disease. *American Journal of Kidney Diseases* 2005;**45**(3):473-84. [MEDLINE: 15754269] |
|  | van den Akker 2003 | an den Akker JM, Bredie SJ, Diepenveen SH, van Tits LJ, Stalenhoef AF, van Leusen R. Atorvastatin and simvastatin in patients on hemodialysis: effects on lipoproteins, C-reactive protein and in vivo oxidized LDL. *Journal of Nephrology* 2003;**16**(2):238-44. [MEDLINE: 12768071] |
|  | Vareesangthip 2005 | Vareesangthip K, Laouthaiwattana P, Hanlakorn P, Suwannaton L, Larpkitkachorn R, Chuawattana D, et al. Effects of simvastatin on the kinetics of erythrocyte sodium lithium countertransport and C-reactive protein in hemodialysis patients [abstract]. *Journal of the American Society of*  *Nephrology* 2005;**16**:276A. |
|  | Velickovic 1997 | Velickovic-Radovanovic R, Avramovic M, Malobabic Z, Djordjevic V, Kostic S, Mitic B. Therapeutic effects of simvastatin on hyperlipidemia in uraemic patients on CAPD [abstract]. *Nephrology Dialysis Transplantation* 1997;**12**(9):A187. |
|  | Vernaglione 2003 | Vernaglione L, Cristofano C, Muscogiuri P, Chimienti S. Does atorvastatin influence serum C-reactive protein levels in patients on long-term hemodialysis?. *American Journal of Kidney Diseases* 2004;**43**(3):471-8. [MEDLINE: 14981605] |
|  | Yu 2007 | Yu MH, Lee JH, Min WK, Chi HS, Chang JW, Yang WS, et al. Effects of ezetimibe and ezetimibe plus simvastatin (Vytorin®) on markers for inflammation and thrombogenesis in end-stage renal disease (ESRD) patients [abstract]. *Nephrology Dialysis Transplantation* 2007;**22**(Suppl 6):vi322. |
| **Kidney transplant recipients (2014)** | | |
|  | ALERT 2001 | Holdaas H, Fellstrom B, Jardine AG, Holme I, Nyberg G, Fauchald P, et al. Effect of fluvastatin on cardiac outcomes in renal transplant recipients: a multicentre, randomised, placebo- controlled trial. *Lancet* 2003;**361**(9374):2024-31. [MEDLINE: 12814712] |
|  | Arnadottir 1994 | Arnadottir M, Eriksson LO, Germershausen JI, Thysell H. Low- dose simvastatin is a well-tolerated and efficacious cholesterol- lowering agent in ciclosporin-treated kidney transplant recipients: double-blind, randomized, placebo-controlled study in 40 patients. *Nephron* 1994;**68**(1):57-62. [MEDLINE: 7991041] |
|  | Bill 1995 | Bill M, Paczek L, Wyzgal J, Baciong Z, Gradowska L, Lao M, Juskowa J, et al. Six-month treatment with low-dose lovastatin does not improve kidney allograft function. *Polish Journal of Immunology* 1995;**20**(4):417-9. |
|  | Castelao 2003 | Castelao AM, Grino JM, Andres E, Gilvernet S, Seron D, Castineiras MJ, et al. HMGCoA reductase inhibitors lovastatin and simvastatin in the treatment of hypercholesterolemia after renal transplantation. *Transplantation Proceedings* 1993;**25**(1 Pt 2):1043-6. [MEDLINE: 8442035] |
|  | Celik 2000 | Celik A, Unsal A, Mutaf I, Habif S, Ok E, Bayindir O. Which dosage of simvastatin in renal transplant patients?. *Nephron* 2000;**84**(1):81-2. [MEDLINE: 10644914] |
|  | Cofan 2002 | Cofan F, Zambon D, Laguna JC, Casals E, Ros E, Cofan M, et al. Pravastatin improves low-density lipoprotein oxidation in renal transplantation. *Transplantation Proceedings* 2002;**34**(2):389-91. [MEDLINE: 11959339] |
|  | Hausberg 2001 | Hausberg M, Kosch M, Stam F, Heidenreich S, Kisters K, Rahn KH, et al. Effect of fluvastatin on endothelium- dependent brachial artery vasodilation in patients after renal transplantation. *Kidney International* 2001;**59**(4):1473-9. [MEDLINE: 11260410] |
|  | Kasiske 2001 | Kasiske BL, Heim-Duthoy KL, Singer GG, Watschinger B, Germain MJ, Bastani B. The effects of lipid-lowering agents on acute renal allograft rejection. *Transplantation* 2001;**72**(2):223-7. [MEDLINE: 11477342] |
|  | Katznelson 1996 | Katznelson S, Wilkinson AH, Kobashigawa JA, Wang XM, Chia D, Ozawa M, et al. The effect of pravastatin on acute rejection after kidney transplantation--a pilot study. *Transplantation* 1996;**61**(10):1469-74. [MEDLINE: 8633373] |
|  | Lepre 1999 | Lepre F, Rigby R, Hawley C, Saltissi D, Brown A, Walsh Z. A double-blind placebo controlled trial of simvastatin for the treatment of dyslipidaemia in renal allograft recipients. *Clinical Transplantation* 1999;**13**(6):520-5. [MEDLINE: 10617243] |
|  | Martinez Hernandez 1993 | Martinez Hernandez BE, Persaud JW, Varghese Z, Moorhead JF. Low-dose simvastatin is safe in hyperlipidaemic renal transplant patients. *Nephrology Dialysis Transplantation* 1993;**8**(7):637-41. [MEDLINE: 8396749] |
|  | Melchor 1998 | Melchor JL, Gracida C. Treatment of hypercholesterolemia with fluvastatin in kidney transplant patients. *Transplantation Proceedings* 1998;**30**(5):2054. [MEDLINE: 9723390] |
|  | Raiola 1998 | Raiola P, Manzo M, Saggese A. Comparison of atorvastatin (ATV) with fluvastatin (FLV) in renal transplant patients with dyslipoproteinemia [abstract]. *Journal of the American Society of Nephrology* 1998;**9**(Program & Abstracts):693A. [CENTRAL: CN-00447330] |
|  | Renders 2001 | Renders L, Mayer-Kadner I, Koch C. Efficacy and drug interactions of the new HMG-CoA reductase inhibitors cerivastatin and atorvastatin in CsA-treated renal transplant recipients. *Nephrology Dialysis Transplantation* 2001;**16**(1):141-6. [MEDLINE: 11209008] |
|  | Sahu 2001 | Sahu K, Sharma R, Gupta A. Effect of lovastatin, an HMG CoA reductase inhibitor, on acute renal allograft rejection. *Clinical Transplantation* 2001;**15**(3):173-5. [MEDLINE: 11389707] |
|  | Santos 2001 | Santos AF, Keitel E, Bittar AE, Neumann J, Fuchs FD, Goldani JC, et al. Safety and efficacy of simvastatin for hyperlipidemia in renal transplant recipients: a double- blind, randomized, placebo-controlled study. *Transplantation Proceedings* 2001;**33**(1-2):1194-5. [MEDLINE: 11267254] |
|  | Seron 2008 | eron D, Oppenheimer F, Pallardo LM, Lauzurica R, Errasti P, Gomez-Huertas E, et al. Fluvastatin in the prevention of renal transplant vasculopathy: results of a prospective, randomized, double-blind, placebo-controlled trial. *Transplantation* 2008;**86**(1):82-7. [MEDLINE: 18622282] |
|  | Sharif 2009 | Sharif A, Ravindran V, Moore R, Dunseath G, Luzio S, Owens D, et al. The effect of rosuvastatin on insulin sensitivity and pancreatic beta-cell function in nondiabetic renal transplant recipients. *American Journal of Transplantation* 2009;**9**(6):1439-45. [MEDLINE: 19459810] |
|  | SOLAR 2001 | Holdaas H, Jardine AG, Wheeler DC, Brekke IB, Conlon PJ, Fellstrom B, et al. Effect of fluvastatin on acute renal allograft rejection: A randomized multicenter trial. *Kidney International* 2001;**60**(5):1990-7. [MEDLINE: 11703619] |
|  | Tuncer 2000 | Tuncer M, Suleymanlar G, Ersoy FF, Yakupoglu G. Comparison of the effects of simvastatin and pravastatin on acute rejection episodes in renal transplant patients. *Transplantation Proceedings* 2000;**32**(3):622-5. [MEDLINE: 10812143] |
|  | UK-HARP-I 2005 | Baigent C, Landray M, Leaper C, Altmann P, Armitage J, Baxter A, et al. First United Kingdom Heart and Renal Protection (UK-HARP-I) study: biochemical efficacy and safety of simvastatin and safety of low-dose aspirin in chronic kidney disease. *American Journal of Kidney Diseases* 2005;**45**(3):473-84. [MEDLINE: 15754269] |
|  | Vergoulas 1999 | Vergoulas G, Miserlis G, Gakis D, Imvrios G, Papagiannis A, Papanikolaou V, et al. Lovastatin (L) versus fluvastatin (F) in the treatment of hypercholesterolemic (HCH) renal transplant recipients (RTR) [abstract]. *Nephrology Dialysis Transplantation* 1999;**14**(9):A312. [CENTRAL: CN-00486302] |
| **Acute coronary syndrome (2014)** | | |
|  | Colivicchi 2002 | Colivicchi F, Guido V, Tubaro M, Ammirati F, Montefoschi N, Varveri A, et al.Effects of atorvastatin 80 mg daily early after onset of unstable angina pectoris or non-Q-wave myocardial infarction. *American Journal of Cardiology* 2002; **90**(8):872-4. |
|  | de Lemos 2004 | de Lemos JA, Blazing MA, Wiviott SD, Lewis EF, Fox KAA, White HD, et al.Early intensive vs a delayed conservative simvastatin strategy in patients with acute coronary syndromes: phase Z of the A to Z trial. *JAMA* 2004;**292** (11):1307–16. |
|  | ESTABLISH 2004 | Okazaki S, Yokoyama T, Miyauchi K, Shimada K, Kurata T, Sato H, et al.Early statin treatment in patients with acute coronary syndrome: demonstration of the beneficial effect on atherosclerotic lesions by serial volumetric intravascular ultrasound analysis during half a year after coronary event: the ESTABLISH Study. *Circulation* 2004;**110**(9):1061–8. |
|  | FACS 2010 | Ostadal P, Alan D, Vejvoda J, Kukacka J, Macek M, Hajek P, et al.Fluvastatin in the first-line therapy of acute coronary syndrome: results of the multicenter, randomized, double- blind, placebo-controlled trial (the FACS-trial). *Trials* 2010;**11**(1):61. |
|  | FLORIDA 2002 | Liem AH, van Boven AJ, Veeger NJGM, Withagen AJ, Robles de Medina RM, Tijssen JGP, et al.Effect of fluvastatin on ischaemia following acute myocardial infarction: a randomized trial. *European Heart Journal* 2002;**23**(24): 1931–7. |
|  | LAMIL 1997 | Kesteloot H, Claeys G, Blanckaert N, Lesaffre E. Time course of serum lipids and apolipoproteins after acute myocardial infarction: modification by pravastatin. *Acta Cardiologica* 1997;**52**(2):107-16. |
|  | L-CAD 2000 | Arntz HR, Agrawal R, Wunderlich W, Schnitzer L, Stern R, Fischer F, et al.Beneficial effects of pravastatin (+/- colestyramine/niacin) initiated immediately after a coronary event (the randomized lipid-coronary artery disease [L- CAD] study). *American Journal of Cardiology* 2000;**86**(12): 1293-8. |
|  | LIPS 2002 | Serruys PW, de Feyter P, Macaya C, Kokott N, Puel J, Vrolix M, et al.Fluvastatin for prevention of cardiac events following successful first percutaneous coronary intervention: a randomized controlled trial. *JAMA* 2002; **287**(24):3215. |
|  | Macin 2005 | Macin SM, Perna ER, Farias EF, Franciosi V, Cialzeta JR, Brizuela M, et al.Atorvastatin has an important acute anti-inflammatory effect in patients with acute coronary syndrome: results of a randomized, double-blind, placebo- controlled study. *American Heart Journal* 2005;**149**(3):451- 7. |
|  | MIRACL 2001 | Schwartz GG, Olsson AG, Ezekowitz MD, Ganz P, Oliver MF, Waters D, et al.Effects of atorvastatin on early recurrent ischemic events in acute coronary syndromes: the MIRACL study: a randomized controlled trial. *JAMA* 2001;**285**(13): 1711. |
|  | OACIS-LIPID 2008 | Sato H, Kinjo K, Ito H, Hirayama A, Nanto S, Fukunami M, et al.Effect of early use of low-dose pravastatin on major adverse cardiac events in patients with acute myocardial infarction: the OACIS-LIPID study. *Circulation* 2008;**72** (1):17. |
|  | PACT 2004 | Thompson PL, Meredith I, Amerena J, Campbell TJ, Sloman JG, Harris PJ. Effect of pravastatin compared with placebo initiated within 24 hours of onset of acute myocardial infarction or unstable angina: the Pravastatin in Acute Coronary Treatment (PACT) trial. *American Heart Journal* 2004;**148**(1):91. |
|  | PAIS 2001 | den Hartog FR, van Kalmthout PM, van Loenhout TT, Schaafsma HJ, Rila H, Verheugt FW. Pravastatin in acute ischaemic syndromes: results of a randomised placebo- controlled trial. *International Journal of Clinical Practice* 2001;**55**(5):300. |
|  | PTT 2002 | Kayikçio lu M, Can L, Kültürsay H, Payzin S, Turko  lu C. Early use of pravastatin in patients with acute myocardial infarction undergoing coronary angioplasty. *Acta Cardiologica* 2002;**57**(4):295. |
|  | RECIFE 1999 | Dupuis J, Tardif JC, Cernacek P, Theroux P. Cholesterol reduction rapidly improves endothelial function after acute coronary syndromes: the RECIFE (reduction of cholesterol in ischemia and function of the endothelium) trial. *Circulation* 1999;**99**(25):3227. |
|  | Ren 2009 | Ren HZ, Ma LL, Wang LX. Effect of simvastatin on plasma interleukin-6 in patients with unstable angina. *Clinical and Investigative Medicine. Médecine Clinique et Experimentale* 2009;**32**(4):E280. |
|  | Sakamoto 2005 | Sakamoto T, Kojima S, Ogawa H, Shimomura H, Kimura K, Ogata Y, et al.Effects of early statin treatment on symptomatic heart failure and ischemic events after acute myocardial infarction in Japanese. *American Journal of Cardiology* 2006;**97**(8):1165-71. |
|  | Shal’nev 2007 | Shal’nev VI. The effects of early application of simvastatin on C-reactive protein level, blood lipids, and the clinical course of acute coronary syndrome. *Klinicheskaia Meditsina* 2007;**85**(11):46–50. |
| **Aortic valve stenosis (2016)** | | |
|  | Chan 2010 | Chan KL, Teo K, Dumesnil JG, Ni A, Tam J, ASTRONOMER Investigators. Effect of lipid lowering with rosuvastatin on progression of aortic stenosis: results of the aortic stenosis progression observation: measuring effects of rosuvastatin (ASTRONOMER) trial. *Circulation* 2010;**121**(2):306-14. |
|  | Cowell 2005 | Cowell SJ, Newby DE, Prescott RJ, Bloomfield P, Reid J, Northridge DB, et al. Scottish Aortic Stenosis and Lipid Lowering Trial, Impact on Regression (SALTIRE) Investigators. A randomized trial of intensive lipid-lowering therapy in calcific aortic stenosis. *New England Journal of Medicine* 2005;**352**:2389-97. |
|  | Rossebø 2008 | Rossebø AB, Pedersen TR, Boman K, Brudi P, Chambers JB, Egstrup K, et al. SEAS Investigators. Intensive lipid lowering with simvastatin and ezetimibe in aortic stenosis. *New England Journal of Medicine* 2008;**359**(13):1343-56. |
|  | van der Linde 2011 | van der Linde D, Yap SC, van Dijk AP, Budts W, Pieper PG, van der Burgh PH, et al. Effects of rosuvastatin on progression of stenosis in adult patients with congenital aortic stenosis (PROCAS Trial). *American Journal of Cardiology* 2011;**108**(2):265-71. |
| **Chronic kidney disease not requiring dialysis (2023)** | | |
|  | 4S 1993 | Chonchol M, Cook T, Kjekshus J, Pedersen TR, Lindenfeld J. Simvastatin for secondary prevention of all-cause mortality and major coronary events in patients with mild chronic renal insufficiency. *American Journal of Kidney Diseases* 2007;**49**(3):373-82. [MEDLINE: 17336698] |
|  | Abe 2011 | Abe M, Maruyama N, Okada K, Matsumoto S, Matsumoto K, Soma M. Effects of lipid-lowering therapy with rosuvastatin on kidney function and oxidative stress in patients with diabetic nephropathy. *Journal of Atherosclerosis & Thrombosis* 2011;**18**(11):1018-28. [MEDLINE: 21921413] |
|  | Abe 2015 | Abe M, Maruyama N, Maruyama T, Okada K, Soma M. A trial of pitavastatin versus rosuvastatin for dyslipidemia in chronic kidney disease. *Journal of Atherosclerosis & Thrombosis* 2015;**22**(12):1235-47. [MEDLINE: 26156625] |
|  | AFCAPS/TexCAPS 1998 | Kendrick J, Shlipak MG, Targher G, Cook T, Lindenfeld J, Chonchol M. Effect of lovastatin on primary prevention of cardiovascular events in mild CKD and kidney function loss: a post hoc analysis of the Air Force/Texas Coronary Atherosclerosis Prevention Study. *American Journal of Kidney Diseases* 2010;**55**(1):42-9. [MEDLINE: 19932541] |
|  | ALLIANCE 2000 | Koren MJ, Davidson MH, Wilson DJ, Fayyad RS, Zuckerman A, Reed DP, et al. Focused atorvastatin therapy in managed-care patients with coronary heart disease and CKD. *American Journal of Kidney Diseases* 2009;**53**(5):741-50. [MEDLINE: 19216014] |
|  | Aranda Arcas 1994 | Aranda Arcas JL, Sanchez R, Guijarro C, Araque A, Pulido F, Praga M, et al. Effect of pravastatin on hypercholesterolemia associated with proteinuria [Efecto de la pravastatina en la hipercolesterolemia asociada a proteinuria]. *Anales de Medicina Interna* 1994;**11**(11):523-7. [MEDLINE: 7654898] |
|  | ASCOT-LLA 2003 | Sever PS, Dahlof B, Poulter NR, Wedel H, Beevers G, Caulfield M, et al. Prevention of coronary and stroke events with atorvastatin in hypertensive patients who have average or lower-than-average cholesterol concentrations, in the Anglo- Scandinavian Cardiac Outcomes Trial--Lipid Lowering Arm (ASCOT-LLA): a multicentre randomised controlled trial. *Lancet* 2003;**361**(9364):1149-58. [MEDLINE: 12686036] |
|  | ASUCA 2013 | Kimura G, Kasahara M, Ueshima K, Tanaka S, Yasuno S, Fujimoto A, et al. Effects of atorvastatin on renal function in patients with dyslipidemia and chronic kidney disease: assessment of clinical usefulness in CKD patients with atorvastatin (ASUCA) trial. *Clinical & Experimental Nephrology* 2017;**21**(3):417-24. [MEDLINE: 27392909] |
|  | Bianchi 2003 | Bianchi S, Bigazzi R, Caiazza A, Campese VM. A controlled, prospective study of the effects of atorvastatin on proteinuria and progression of kidney disease [Erratum in: Am J Kidney Dis. 2004 Jan;43(1):193]. *American Journal of Kidney Diseases* 2003;**41**(3):565-70. [MEDLINE: 12612979] |
|  | Buemi 2000 | Buemi M, Allegra A, Corica F, Aloisi C, Giacobbe M, Pettinato G, et al. Effect of fluvastatin on proteinuria in patients with immunoglobulin A nephropathy. *Clinical Pharmacology & Therapeutics* 2000;**67**(4):427-31. [MEDLINE: 10801253] |
|  | CARDS 2003 | Colhoun HM, Betteridge DJ, Durrington PN, Hitman GA, Neil HA, Livingstone SJ, et al. Effects of atorvastatin on kidney outcomes and cardiovascular disease in patients with diabetes: an analysis from the Collaborative Atorvastatin Diabetes Study (CARDS). *American Journal of Kidney Diseases* 2009;**54**(5):810-9. [MEDLINE: 19540640] |
|  | Cha 2015 | Cha JJ, Kim K, Min HS, Ghee J, Kim YJ, Lee EY, et al. Effect of fluvastatin treatment on proteinuria in diabetic patients with chronic kidney disease [abstract no: TH-PO656]. *Journal of the American Society of Nephrology* 2015;**26**(Abstract Suppl):239a. [EMBASE: 641102077] |
|  | Di Lullo 2005 | Di Lullo L, Addesse R, Comegna C, Firmi G, Galderisi C, Iannacci GR, et al. Effects of fluvastatin treatment on lipid profile, C-reactive protein trend, and renal function in dyslipidemic patients with chronic renal failure. *Advances in Therapy* 2005;**22**(6):601-12. [MEDLINE: 16510377] |
|  | Dummer 2008 | Dummer CD, Thome' FS, Zingano B, Lindoso A, Veronese FV. Acute effect of simvastatin on inflammation and oxidative stress in chronic kidney disease. *Journal of Nephrology* 2008;**21**(6):900-8. [MEDLINE: 19034875] |
|  | ESPLANADE 2010 | Ruggenenti P, Perna A, Tonelli M, Loriga G, Motterlini N, Rubis N, et al. Effects of add-on fluvastatin therapy in patients with chronic proteinuric nephropathy on dual renin-angiotensin system blockade: the ESPLANADE Trial. *Clinical Journal of the American Society of Nephrology: CJASN* 2010;**5**(11):1928-38. [MEDLINE: 20671225] |
|  | Fassett 2010 | Fassett RG, Coombes JS, Packham D, Fairley KF, Kincaid- Smith P. Effect of pravastatin on kidney function and urinary protein excretion in autosomal dominant polycystic kidney disease. *Scandinavian Journal of Urology & Nephrology* 2010;**44**(1):56-61. [MEDLINE: 20034362] |
|  | Fried 2001 | Fried LF, Forrest KY, Ellis D, Chang Y, Silvers N, Orchard TJ. Lipid modulation in insulin-dependent diabetes mellitus: effect on microvascular outcomes. *Journal of Diabetes & its Complications* 2001;**15**(3):113-9. [MEDLINE: 11358679] |
|  | Gheith 2002 | Gheith OA, Sobh MA, Mohamed Kel-E, El-Baz M, El-Husseini F, Gazarin SS, et al. Impact of treatment of dyslipidemia on renal function, fat deposits and scarring in patients with persistent nephrotic syndrome. *Nephron* 2002;**91**(4):612-9. [MEDLINE: 12138263] |
|  | Goicoechea 2006 | Goicoechea M, de Vinuesa SG, Lahera V, Cachofeiro V, Gomez-Campdera F, Vega A, et al. Effects of atorvastatin on inflammatory and fibrinolytic parameters in patients with chronic kidney disease. *Journal of the American Society of Nephrology* 2006;**17**(12 Suppl 3):S231-5. [MEDLINE: 17130267] |
|  | Hommel 1992 | Hommel E, Andersen P, Gall MA, Nielsen F, Jensen B, Rossing P, et al. Plasma lipoproteins and renal function during simvastatin treatment in diabetic nephropathy. *Diabetologia* 1992;**35**(5):447-51. [MEDLINE: 1521727] |
|  | HPS 2002 | Collins R, Armitage J, Parish S, Sleigh P, Peto R. MRC/BHF Heart Protection Study of cholesterol-lowering with simvastatin in 5963 people with diabetes: a randomised placebo-controlled trial. *Lancet* 2003;**361**(9374):2005-16. [MEDLINE: 12814710] |
|  | IDEAL 2004 | Holme I, Fayyad R, Faergeman O, Kastelein JJ, Olsson AG, Tikkanen MJ, et al. Cardiovascular outcomes and their relationships to lipoprotein components in patients with and without chronic kidney disease: results from the IDEAL trial. *Journal of Internal Medicine* 2010;**267**(6):567-75. [MEDLINE: 20141566] |
|  | Ikeda 2012 | Ikeda H, Ura Y, Nakayama M. Rosuvastatin reduces urinary protein excretion in patients with chronic glomerulonephritis compared to the pravastatin use: a crossover trial [abstract no: SA-PO274]. *Journal of the American Society of Nephrology* 2012;**23**(Abstract Suppl):699A. |
|  | Imai 1999 | Imai Y, Suzuki H, Saito T, Tsuji I, Abe K, Saruta T. The effect of pravastatin on renal function and lipid metabolism in patients with renal dysfunction with hypertension and hyperlipidemia. Pravastatin and Renal Function Research Group. *Clinical & Experimental Hypertension (New York)* 1999;**21**(8):1345-55. [MEDLINE: 10574417] |
|  | Inukai 2011 | Inukai K, Iuchi T, Sumita T, Ito D, Ikebukuro K, Imai K, et al. Combination therapy with angiotensin receptor blocker (ARB) plus atorvastatin reduced proteinuria in type 2 diabetic patients with diabetic nephropathy. *Therapeutic Research* 2011;**32**(7):947-53. [EMBASE: 362412882] |
|  | JUPITER 2007 | Ridker PM, MacFadyen J, Cressman M, Glynn RJ. Efficacy of rosuvastatin among men and women with moderate chronic kidney disease and elevated high-sensitivity C-reactive protein: a secondary analysis from the JUPITER (Justification for the Use of Statins in Prevention-an Intervention Trial Evaluating Rosuvastatin) trial. *Journal of the American College of Cardiology* 2010;**55**(12):1266-73. [MEDLINE: 20206456] |
|  | Kimura 2012 | Kimura S, Inoguchi T, Yokomizo H, Maeda Y, Sonoda N, Takayanagi R. Randomized comparison of pitavastatin and pravastatin treatment on the reduction of urinary albumin in patients with type 2 diabetic nephropathy. *Diabetes, Obesity & Metabolism* 2012;**14**(7):666-9. [MEDLINE: 22268518] |
|  | Lam 1995 | Lam KS, Cheng IK, Janus ED, Pang RW. Cholesterol-lowering therapy may retard the progression of diabetic nephropathy. *Diabetologia* 1995;**38**(5):604-9. [MEDLINE: 7489845] |
|  | Lee 2002 | Lee TM, Lin MS, Tsai CH, Chang NC. Add-on and withdrawal effect of pravastatin on proteinuria in hypertensive patients treated with AT receptor blockers. *Kidney International* 2005;**68**(2):779-87. [MEDLINE: 16014056] |
|  | Lintott 1995 | Lintott CJ, Scott RS, Bremer JM, Shand BI. Fluvastatin for dyslipoproteinemia, with or without concomitant chronic renal insufficiency. *American Journal of Cardiology* 1995;**76**(2):97-101A. [MEDLINE: 7604809] |
|  | LIPS 2001 | Lemos PA, Serruys PW, de Feyter P, Mercado NF, Goedhart D, Saia F, et al. Long-term fluvastatin reduces the hazardous effect of renal impairment on four-year atherosclerotic outcomes (a LIPS substudy). *American Journal of Cardiology* 2005;**95**(4):445-51. [MEDLINE: 15695126] |
|  | LORD 2006 | Fassett RG, Robertson IK, Ball MJ, Geraghty DP, Coombes JS. Effect of atorvastatin on kidney function in chronic kidney disease: a randomised double-blind placebo-controlled trial. *Atherosclerosis* 2010;**213**(1):218-24. [MEDLINE: 20810109] |
|  | Masajtis-Zagajewska 2018 | Masajtis-Zagajewska A, Nowicki M. Effect of atorvastatin on iron metabolism regulation in patients with chronic kidney disease - a randomized double blind crossover study. *Renal Failure* 2018;**40**(1):700-9. [MEDLINE: 30741616] |
|  | MEGA 2004 | Nakamura H, Mizuno K, Ohashi Y, Yoshida T, Hirao K, Uchida Y, et al. Pravastatin and cardiovascular risk in moderate chronic kidney disease. *Atherosclerosis* 2009;**206**(2):512-7. [MEDLINE: 19423108] |
|  | Mori 1992 | Mori Y, Tsuruoka A. Effect of pravastatin on microalbuminuria in patients with non-insulin-dependent diabetes mellitus. *Journal of the Japan Diabetes Society* 1992;**35**(3):265-8. [EMBASE: 22147775] |
|  | Nakamura 2002 | Nakamura T, Ushiyama C, Hirokawa K, Osada S, Inoue T, Shimada N, et al. Effect of cerivastatin on proteinuria and urinary podocytes in patients with chronic glomerulonephritis. *Nephrology Dialysis Transplantation* 2002;**17**(5):798-802. [MEDLINE: 11981066] |
|  | Nakamura 2005 | Nakamura T, Sugaya T, Kawagoe Y, Ueda Y, Osada S, Koide H. Effect of pitavastatin on urinary liver-type fatty acid-binding protein levels in patients with early diabetic nephropathy. *Diabetes Care* 2005;**28**(11):2728-32. [MEDLINE: 16249547] |
|  | Nakamura 2006 | Nakamura T, Sugaya T, Kawagoe Y, Suzuki T, Inoue T, Node K. Effect of pitavastatin on urinary liver-type fatty-acid-binding protein in patients with nondiabetic mild chronic kidney disease. *American Journal of Nephrology* 2006;**26**(1):82-6. [MEDLINE: 16534182] |
|  | Nielsen 1993 | Nielsen S, Schmitz O, Moller N, Porksen N, Klausen IC, Alberti KG, et al. Renal function and insulin sensitivity during simvastatin treatment in type 2 (non-insulin-dependent) diabetic patients with microalbuminuria. *Diabetologia* 1993;**36**(10):1079-86. [MEDLINE: 8243858] |
|  | Ohsawa 2015 | Ohsawa M, Tamura K, Wakui H, Kanaoka T, Azushima K, Uneda K, et al. Effects of pitavastatin add-on therapy on chronic kidney disease with albuminuria and dyslipidemia. *Lipids in Health & Disease* 2015;**14**:161. [MEDLINE: 26645467] |
|  | PANDA 2011 | Rutter MK, Prais HR, Charlton-Menys V, Gittins M, Roberts C, Davies RR, et al. Protection Against Nephropathy in Diabetes with Atorvastatin (PANDA): a randomized double-blind placebo- controlled trial of high- vs. low-dose atorvastatin. *Diabetic Medicine* 2011;**28**(1):100-8. [MEDLINE: 21166851] |
|  | Panichi 2006 | Panichi V, Paoletti S, Mantuano E, Manca-Rizza G, Filippi C, Santi S, et al. In vivo and in vitro effects of simvastatin on inflammatory markers in pre-dialysis patients. *Nephrology Dialysis Transplantation* 2006;**21**(2):337-44. [MEDLINE: 16249194] |
|  | PLANET I 2006 | de Zeeuw D, Anzalone DA, Cain VA, Cressman MD, Heerspink HJ, Molitoris BA, et al. Renal effects of atorvastatin and rosuvastatin in patients with diabetes who have progressive renal disease (PLANET I): a randomised clinical trial. *The Lancet Diabetes & Endocrinology* 2015;**3**(3):181-90. [MEDLINE: 25660356] |
|  | PLANET II 2006 | Idzerda NM, Pena MJ, Parving HH, de Zeeuw D, Heerspink HJ. Proteinuria and cholesterol reduction are independently associated with less renal function decline in statin-treated patients; a post hoc analysis of the PLANET trials. *Nephrology Dialysis Transplantation* 2019;**34**(10):1699-706. [MEDLINE:  30184238]  Kroonen M, Stevens J, De Zeeuw D, Heerspink HJL. Association between individual cholesterol and albuminuria response and exposure to atorvastatin or rosuvastatin [abstract no: FR-PO253]. *Journal of the American Society of Nephrology* 2019;**30**(Abstract Suppl):500. [EMBASE: 633769807] |
|  | PPP 1992 | Tonelli M, Isles C, Curhan GC, Tonkin A, Pfeffer MA, Shepherd J, et al. Effect of pravastatin on cardiovascular events in people with chronic kidney disease. *Circulation* 2004;**110**(12):1557-63. [MEDLINE: 15364796] |
|  | PREVEND IT 2000 | Asselbergs FW, Diercks GF, Hillege HL, van Boven AJ, Janssen WM, Voors AA, et al. Effects of fosinopril and pravastatin on cardiovascular events in subjects with microalbuminuria. *Circulation* 2004;**110**(18):2809-16. [MEDLINE: 15492322] |
|  | Rayner 1996 | Rayner BL, Byrne MJ, van Zyl Smit R. A prospective clinical trial comparing the treatment of idiopathic membranous nephropathy and nephrotic syndrome with simvastatin and diet, versus diet alone. *Clinical Nephrology* 1996;**46**(4):219-24. [MEDLINE: 8905205] |
|  | Renke 2010 | Renke M, Tylicki L, Rutkowski P, Neuwelt A, Larczynski W, Zietkiewicz M, et al. Atorvastatin improves tubular status in non-diabetic patients with chronic kidney disease – placebo ontrolled, randomized, cross-over study. *Acta Biochimica Polonica* 2010;**57**(4):547-52. [MEDLINE: 21079818] |
|  | SAGE 2004 | Deedwania P, Stone PH, Bairey Merz CN, Cosin-Aguilar J, Koylan N, Luo D, et al. Effects of intensive versus moderate lipid- lowering therapy on myocardial ischemia in older patients with coronary heart disease: results of the Study Assessing Goals in the Elderly (SAGE). *Circulation* 2007;**115**(6):700-7. [MEDLINE: 17283260] |
|  | Samuelsson 2002 | Samuelsson O, Attman PO, Knight-Gibson C, Mulec H, Weiss L, Alaupovic P. Fluvastatin improves lipid abnormalities in patients with moderate to advanced chronic renal insufficiency. *American Journal of Kidney Diseases* 2002;**39**(1):67-75. [MEDLINE: 11774104] |
|  | Sawara 2008 | Sawara Y, Takei T, Uchida K, Ogawa T, Yoshida T, Tsuchiya K, et al. Effects of lipid-lowering therapy with rosuvastatin on atherosclerotic burden in patients with chronic kidney disease. *Internal Medicine* 2008;**47**(17):1505-10. [MEDLINE: 18758125] |
|  | Scanferla 1991 | Scanferla F, Toffoletto PP, Roncali D, Bazzato G. Associated effect of hepatic hydroxymethylglutaryl coenzyme A reductase + angiotensin converting enzyme inhibitors on the progression of renal failure in hypertensive subjects. *American Journal of Hypertension* 1991;**4**(10 Pt 1):868-74. [MEDLINE: 1747221] |
|  | SHARP 2010 | Baigent C, Landray MJ, Reith C, Emberson J, Wheeler DC, Tomson C, et al. The effects of lowering LDL cholesterol with simvastatin plus ezetimibe in patients with chronic kidney disease (Study of Heart and Renal Protection): a randomised placebo-controlled trial. *Lancet* 2011;**377**(9784):2181-92. [PMID: 21663949] |
|  | Stegmayr 2005 | Holmberg B, Brannstrom M, Bucht B, Crougneau V, Dimeny E, Ekspong A, et al. Safety and efficacy of atorvastatin in patients with severe renal dysfunction. *Scandinavian Journal of Urology & Nephrology* 2005;**39**(6):503-10. [MEDLINE: 16303728] |
|  | Thomas 1993 | Thomas ME, Harris KP, Ramaswamy C, Hattersley JM, Wheeler DC, Varghese Z, et al. Simvastatin therapy for hypercholesterolemic patients with nephrotic syndrome or significant proteinuria. *Kidney International* 1993;**44**(5):1124-9. [MEDLINE: 8264145] |
|  | TNT 2004 | Shepherd J, Kastelein JJ, Bittner V, Deedwania P, Breazna A, Dobson S, et al. Intensive lipid lowering with atorvastatin in patients with coronary heart disease and chronic kidney disease: the TNT (Treating to New Targets) study. *Journal of the American College of Cardiology* 2008;**51**(15):1448-54. [MEDLINE: 18402899] |
|  | Tokunaga 2008 | Tokunaga M, Tamura M, Kabashima N, Serino R, Shibata T, Matsumoto M, et al. Beneficial effects of pitavastatin on albuminuria and renal function in essential hypertensive patients with chronic kidney disease [abstract no: TH-PO875]. *Journal of the American Society of Nephrology* 2008;**19**(Abstracts Issue):307A. [CENTRAL: CN-00773767] |
|  | Tonolo 1997 | Tonolo G, Ciccarese M, Brizzi P, Puddu L, Secchi G, Calvia P, et al. Reduction of albumin excretion rate in normotensive microalbuminuric type 2 diabetic patients during long-term simvastatin treatment. *Diabetes Care* 1997;**20**(12):1891-5. [MEDLINE: 9405913] |
|  | UK-HARP-I 2005 | Baigent C, Landray M, Leaper C, Altmann P, Armitage J, Baxter A, et al. First United Kingdom Heart and Renal Protection (UK-HARP-I) study: biochemical efficacy and safety of simvastatin and safety of low-dose aspirin in chronic kidney disease. *American Journal of Kidney Diseases* 2005;**45**(3):473-84. [MEDLINE: 15754269] |
|  | Verma 2005 | Verma A, Ranganna KM, Reddy RS, Verma M, Gordon NF. Effect of rosuvastatin on C-reactive protein and renal function in patients with chronic kidney disease. *American Journal of Cardiology* 2005;**96**(9):1290-2. [MEDLINE: 16253600] |
|  | Yasuda 2004 | Yasuda G, Kuji T, Hasegawa K, Ogawa N, Shimura G, Ando D, et al. Safety and efficacy of fluvastatin in hyperlipidemic patients with chronic renal disease. *Renal Failure* 2004;**26**(4):411-8. [MEDLINE: 15462110] |
|  | Yi 2014 | Yi YJ, Kim HJ, Jo SK, Kim SG, Song YR, Chung W, et al. Comparison of the efficacy and safety profile of morning administration of controlled-release simvastatin versus evening administration of immediate-release simvastatin in chronic kidney disease patients with dyslipidemia. *Clinical Therapeutics* 2014;**36**(8):1182-90. [MEDLINE: 24996489] |
|  | Zhang 1995 | Zhang A, Vertommen J, Van Gaal L, De Leeuw I. Effects of pravastatin on lipid levels, in vitro oxidizability of non-HDL lipoproteins and microalbuminuria in IDDM patients. *Diabetes Research & Clinical Practice* 1995;**29**(3):189-94. [MEDLINE: 8591712] |

| eTable 2. List of studies in each Cochrane SGLT-2 inhibitor Review | | |
| --- | --- | --- |
|  | **Trial** | **Reference** |
| **Cardiovascular Disease (2021)** | | |
|  | Bhatt 2021 | Bhatt DL, Szarek M, Steg PG, Cannon CP, Leiter LA, McGuire DK, et al, SOLOIST-WHF Trial Investigators. Sotagliflozin In patients with diabetes and recent worsening heart failure. *New England Journal of Medicine* 2021;**384**(2):117-28. [PMID: 33200892] |
|  | Cefalu 2015 | Cefalu WT, Leiter LA, de Bruin TW, Gause-Nilsson I, Sugg J, Parikh SJ. Dapagliflozin's effects on glycemia and cardiovascular risk factors in high-risk patients with type 2 diabetes: a 24-week, multicenter, randomized, double-blind, placebo-controlled study with a 28-week extension. *Diabetes Care* 2015;**38**(7):1218-27. |
|  | Cannon 2020 | annon CP, Pratley R, Dagogo-Jack S, Mancuso J, Huyck S, Masiukiewicz U, et al, VERTIS CV Investigators. Cardiovascular outcomes with ertugliflozin in type 2 diabetes. *New England Journal of Medicine* 2020;**383**(15):1425-35. [PMID: 32966714] |
|  | McMurray 2019 | McMurray JJ, Solomon SD, Inzucchi SE, Køber L, Kosiborod MN, Martinez FA, et al. Dapagliflozin in patients with heart failure and reduced ejection fraction. *New England Journal of Medicine* 2019;**381**(21):1995-2008. |
|  | Packer 2020 | Packer M, Anker SD, Butler J, Filippatos G, Pocock SJ, Carson P, et al, EMPEROR-Reduced Trial Investigators. Cardiovascular and renal outcomes with empagliflozin in heart failure. *New England Journal of Medicine* 2020;**383**(15):1413-24. [PMID: 32865377] |
|  | Leiter 2014 | Leiter LA, Cefalu WT, de Bruin TW, Gause-Nilsson I, Sugg J, Parikh SJ. Dapagliflozin added to usual care in individuals with type 2 diabetes mellitus with preexisting cardiovascular disease: a 24-week, multicenter, randomized, double-blind, placebo-controlled study with a 28-week extension. *Journal of the American Geriatrics Society* 2014;**62**(7):1252-62. |
|  | Neal 2017 | Neal B, Perkovic V, Mahaffey KW, de Zeeuw D, Fulcher G, Erondu N, et al. Canagliflozin and cardiovascular and renal events in type 2 diabetes. *New England Journal of Medicine* 2017;**377**(7):644-57. |
|  | Phrommintikul 2019 | Phrommintikul A, Wongcharoen W, Kumfu S, Jaiwongkam T, Gunaparn S, Chattipakorn S, et al. Effects of dapagliflozin vs vildagliptin on cardiometabolic parameters in diabetic patients with coronary artery disease: a randomised study. *British Journal of Clinical Pharmacology* 2019;**85**(6):1337-47. |
|  | Shimizu 2020 | Shimizu W, Kubota Y, Hoshika Y, Mozawa K, Tara S, Tokita Y, et al. Effects of empagliflozin versus placebo on cardiac sympathetic activity in acute myocardial infarction patients with type 2 diabetes mellitus: the EMBODY trial. *Cardiovascular Diabetology* 2020;**19**(1):148. |
|  | Tanaka 2019 | Tanaka A, Shimabukuro M, Machii N, Teragawa H, Okada Y, Shima KR, et al. Effect of empagliflozin on endothelial function in patients with type 2 diabetes and cardiovascular disease: results from the multicenter, randomized, placebo-controlled, double-blind EMBLEM trial. *Diabetes Care* 2019;**42**(10):e159-61. |
|  | Tanaka 2020 | Tanaka A, Hisauchi I, Taguchi I, Sezai A, Toyoda S, Tomiyama H, et al. Effects of canagliflozin in patients with type 2 diabetes and chronic heart failure: a randomized trial (CANDLE). *ESC Heart Failure* 2020;**7**(4):1585-94. |
|  | Verma 2019 | Verma S, Mazer CD, Yan AT, Mason T, Garg V, Teoh H, et al. Effect of empagliflozin on left ventricular mass in patients with type 2 diabetes mellitus and coronary artery disease: the EMPA-HEART CardioLink-6 randomized clinical trial. *Circulation* 2019;**140**(21):1693-702. |
|  | Zinman 2015 | Zinman B, Wanner C, Lachin JM, Fitchett D, Bluhmki E, Hantel S, et al, EMPA-REG OUTCOME Investigators. Empagliflozin, cardiovascular outcomes, and mortality in type 2 diabetes. *New England Journal of Medicine* 2015;**373**(22):2117-28. |
| **Chronic kidney disease and Diabetes mellitus (2024)** | | |
|  | Allegretti 2019 | Allegretti AS, Zhang W, Zhou W, Thurber TK, Rigby SP, Bowman-Stroud C, et al. Safety and effectiveness of bexagliflozin in patients with type 2 diabetes mellitus and stage 3a/3b CKD. *American Journal of Kidney Diseases* 2019;**74**(3):328-33. [MEDLINE: 31101403] |
|  | CANVAS Program 2017 | Neal B, Perkovic V, Mahaffey KW, de Zeeuw D, Fulcher G, Erondu N, et al. Canagliflozin and cardiovascular and renal events in type 2 diabetes. *New England Journal of Medicine* 2017;**377**(7):644-57. [MEDLINE: 28605608] |
|  | Cherney 2021 | Cherney DZ, Ferrannini E, Umpierrez GE, Peters AL, Rosenstock J, Carroll AK, et al. Efficacy and safety of sotagliflozin in patients with type 2 diabetes and severe renal impairment. *Diabetes Obesity Metabolism* 2021;**23**(12):2632-42. [PMID: 34338408] |
|  | CompoSIT-R 2018 | Scott R, Morgan J, Zimmer Z, Lam RL, O'Neill EA, Kaufman KD, et al. A randomized clinical trial of the efficacy and safety of sitagliptin compared with dapagliflozin in patients with type 2 diabetes mellitus and mild renal insufficiency: The CompoSIT- R study. *Diabetes, Obesity & Metabolism* 2018;**20**(12):2876-84. [PMID: 30019498] |
|  | CREDENCE 2017 | Perkovic V, Jardine MJ, Neal B, Bompoint S, Heerspink HJL, Charytan DM, et al. Canagliflozin and renal outcomes in type 2 diabetes and nephropathy. *New England Journal of Medicine* 2019;**380**(24):2295-306. [MEDLINE: 30990260] |
|  | DAPA-CKD 2020 | Wheeler DC, Stefansson BV, Jongs N, Chertow GM, Greene T, Hou FF, et al. Effects of dapagliflozin on major adverse kidney and cardiovascular events in patients with diabetic and non- diabetic chronic kidney disease: a prespecified analysis from the DAPA-CKD trial. *The Lancet Diabetes & Endocrinology* 2021;**9**(1):22-31. [PMID: 33338413] |
|  | DAPA-HF 2019 | hund PS, Solomon SD, Docherty KF, Heerspink HJL, Anand IS, Bohm M, et al. Efficacy of dapagliflozin on renal function and outcomes in patients with heart failure with reduced ejection fraction: results of DAPA-HF. *Circulation* 2021;**143**(4):298-309. [PMID: 33040613] |
|  | DARE-19 2021 | Kosiborod MN, Esterline R, Furtado RH, Oscarsson J, Gasparyan SB, Koch GG, et al. Dapagliflozin in patients with cardiometabolic risk factors hospitalised with COVID-19 (DARE-19): a randomised, double-blind, placebo- controlled, phase 3 trial. *The Lancet Diabetes & Endocrinology* 2021;**9**(9):586-94. [PMID: 34302745] |
|  | DECLARE-TIMI 58 2018 | Bajaj HS, Raz I, Mosenzon O, Murphy SA, Rozenberg A, Yanuv I, et al. Cardiovascular and renal benefits of dapagliflozin in patients with short and long-standing type 2 diabetes: Analysis from the DECLARE-TIMI 58 trial. *Diabetes Obesity & Metabolism* 2020;**22**(7):1122-31. [PMID: 32090404]  Bonaca MP, Wiviott SD, Zelniker TA, Mosenzon O, Bhatt DL, Leiter LA, et al. Dapagliflozin and cardiac, kidney, and limb outcomes in patients with and without peripheral artery disease in DECLARE-TIMI 58. *Circulation* 2020;**142**(8):734-47. [MEDLINE: 32795086]  Cahn A, Mosenzon O, Wiviott SD, Rozenberg A, Yanuv I, Goodrich EL, et al. Efficacy and safety of dapagliflozin in the elderly: analysis from the DECLARE-TIMI 58 study. *Diabetes Care* 2020;**43**(2):468-75. [MEDLINE: 31843945]  Cahn A, Mosenzon O, Wiviott SD, Rozenberg A, Yanuv I, Goodrich EL, et al. Efficacy and safety of dapagliflozin in the elderly: analysis from the DECLARE-TIMI 58 study. *Diabetes Care* 2020;**43**(2):468-75. [PMID: 31843945] |
|  | DELIGHT 2019 | Pollock C, Stefansson B, Reyner D, Rossing P, Sjostrom CD, Wheeler DC, et al. Albuminuria-lowering effect of dapagliflozin alone and in combination with saxagliptin and effect of dapagliflozin and saxagliptin on glycaemic control in patients with type 2 diabetes and chronic kidney disease (DELIGHT):  a randomised, double-blind, placebo-controlled trial. *The Lancet Diabetes & Endocrinology* 2019;**7**(6):429-41. [MEDLINE: 30992195] |
|  | DEPICT-1 2017 | Dandona P, Mathieu C, Phillip M, Hansen L, Griffen SC, Tschope D, et al. Efficacy and safety of dapagliflozin in patients with inadequately controlled type 1 diabetes (DEPICT-1): 24 week results from a multicentre, double-blind, phase 3, randomised controlled trial. *The Lancet Diabetes & Endocrinology* 2017;**5**(11):864-76. [MEDLINE: 28919061]  Groop PH, Dandona P, Phillip M, Gillard P, Edelman S, Jendle J, et al. Effect of dapagliflozin as an adjunct to insulin over 52 weeks in individuals with type 1 diabetes: post-hoc renal analysis of the DEPICT randomised controlled trials. *The Lancet Diabetes & Endocrinology* 2020;**8**(10):845-54. [MEDLINE: 32946821] |
|  | DEPICT-2 2017 | Mathieu C, Dandona P, Gillard P, Senior P, Hasslacher C, Araki E, et al. Efficacy and safety of dapagliflozin in patients with inadequately controlled type 1 diabetes (the DEPICT-2 Study): 24-week results from a randomized controlled trial. *Diabetes Care* 2018;**41**(9):1938-46. [MEDLINE: 30026335]  Groop PH, Dandona P, Phillip M, Gillard P, Edelman S, Jendle J, et al. Effect of dapagliflozin as an adjunct to insulin over 52 weeks in individuals with type 1 diabetes: post-hoc renal analysis of the DEPICT randomised controlled trials. *The Lancet Diabetes & Endocrinology* 2020;**8**(10):845-54. [MEDLINE: 32946821] |
|  | DERIVE 2018 | Fioretto P, Del Prato S, Buse JB, Goldenberg R, Giorgino F, Reyner D, et al. Efficacy and safety of dapagliflozin in patients with type 2 diabetes and moderate renal impairment (chronic kidney disease stage 3A): The DERIVE Study. *Diabetes, Obesity & Metabolism* 2018;**20**(11):2532-40. [MEDLINE: 29888547] |
|  | DIA3004 2013 | Yale JF, Bakris G, Cariou B, Nieto J, David-Neto E, Yue D, et al. Efficacy and safety of canagliflozin over 52 weeks in patients with type 2 diabetes mellitus and chronic kidney disease. *Diabetes, Obesity & Metabolism* 2014;**16**(10):1016-27. [MEDLINE: 24965700] |
|  | Eickhoff 2018 | Eickhoff M, Frimodt-Moller M, Rye JN, Rossing P, Persson F. Effect of dapagliflozin on albuminuria and the renin-angiotensin system when added to renin-angiotensin blockade in patients with type 2 diabetes and nephropathy [abstract no: SP417]. *Nephrology Dialysis Transplantation* 2018;**33**(Suppl 1):i488. [EMBASE: 622605920] |
|  | EMPA-Kidney 2023 | Herrington WG, Staplin N, Wanner C, Green JB, Hauske SJ, Emberson JR, et al. Empagliflozin in patients with chronic kidney disease. *New England Journal of Medicine* 2023;**388**(2):117-27. [MEDLINE: 36331190] |
|  | EMPA-REG BP 2015 | Tikkanen I, Narko K, Zeller C, Green A, Salsali A, Broedl UC, et al. Empagliflozin reduces blood pressure in patients with type 2 diabetes and hypertension. *Diabetes Care* 2015;**38**(3):420-8. [MEDLINE: 25271206] |
|  | EMPA-REG MET 2013 | Haring HU, Merker L, Seewaldt-Becker E, Weimer M, Meinicke T, Broedl UC, et al. Empagliflozin as add-on to metformin in patients with type 2 diabetes: a 24-week, randomized, double-blind, placebo-controlled trial. *Diabetes Care* 2014;**37**(6):1650-9. [MEDLINE: 24722494] |
|  | EMPA-REG MONO 2013 | Roden M, Weng J, Eilbracht J, Delafont B, Kim G, Woerle HJ, et al. Empagliflozin monotherapy with sitagliptin as an active comparator in patients with type 2 diabetes: a randomised, double-blind, placebo-controlled, phase 3 trial. *The Lancet Diabetes & Endocrinology* 2013;**1**(3):208-19. [MEDLINE: 24622369] |
|  | EMPA-REG OUTCOME 2013 | Wanner C, Lachin JM, Inzucchi SE, Fitchett D, Mattheus M, George JT, et al. Empagliflozin and clinical outcomes in patients with type 2 diabetes mellitus, established cardiovascular disease and chronic kidney disease. *Circulation* 2018;**137**(2):119-29. [MEDLINE: 28904068] |
|  | EMPA-REG PIO 2014 | Kovacs CS, Seshiah V, Swallow R, Jones R, Rattunde H, Woerle HJ, et al. Empagliflozin improves glycaemic and weight control as add-on therapy to pioglitazone or pioglitazone plus metformin in patients with type 2 diabetes: a 24-week, randomized, placebo-controlled trial. *Diabetes, Obesity & Metabolism* 2014;**16**(2):147-58. [MEDLINE: 23906415] |
|  | EMPA-REG RENAL 2014 | Barnett AH, Mithal A, Manassie J, Jones R, Rattunde H, Woerle HJ, et al. Efficacy and safety of empagliflozin added to existing antidiabetes treatment in patients with type 2 diabetes and chronic kidney disease: a randomised, double-blind, placebo-controlled trial. *The Lancet Diabetes & Endocrinology* 2014;**2**(5):369-84. [MEDLINE: 24795251] |
|  | EMPEROR-Preserved 2019 | Anker SD, Butler J, Filippatos G, Ferreira JP, Bocchi E, Böhm M, et al. Empagliflozin in heart failure with a preserved ejection fraction. *New England Journal of Medicine* 2021;**385**(16):1451-61. [PMID: 34449189] |
|  | EMPEROR-Reduced 2020 | Zannad F, Ferreira JP, Pocock SJ, Zeller C, Anker SD, Butler J, et al. Cardiac and kidney benefits of empagliflozin in heart failure across the spectrum of kidney function: insights from EMPEROR-Reduced. *Circulation* 2021;**143**(4):310-21. [PMID: 33095032] |
|  | EMPRA 2022 | Antlanger M, Domenig O, Kaltenecker CC, Kovarik JJ, Rathkolb V, Muller MM, et al. Combined sodium glucose co- transporter-2 inhibitor and angiotensin-converting enzyme inhibition upregulates the renin-angiotensin system in chronic kidney disease with type 2 diabetes: results of a randomized, double-blind, placebo-controlled exploratory trial. *Diabetes, Obesity & Metabolism* 2022;**24**(5):816-26. [PMID: 34984822] |
|  | Haneda 2016 | Haneda M, Seino Y, Inagaki N, Kaku K, Sasaki T, Fukatsu A, et al. Influence of renal function on the 52-week efficacy and safety of the sodium glucose cotransporter 2 inhibitor luseogliflozin in Japanese patients with type 2 diabetes mellitus. *Clinical Therapeutics* 2016;**38**(1):66-88. [MEDLINE: 26718606] |
|  | He 2021 | He Y, Pachori A, Chen P, Ma S, Mendonza AE, Amer A, et al. Glucosuric, renal and haemodynamic effects of licogliflozin, a dual inhibitor of sodium-glucose co-transporter-1 and sodium- glucose co-transporter-2, in patients with chronic kidney disease: a randomized trial. *Diabetes, Obesity and Metabolism* 2021;**23**(5):1182-90. [MEDLINE: 33512754] |
|  | Ikeda 2019 | Ikeda S, Takano Y, Schwab D, Portron A, Kasahara- Ito N, Saito T, et al. Effect of renal impairment on the pharmacokinetics and pharmacodynamics of tofogliflozin (a selective SGLT2 Inhibitor) in patients with type 2 diabetes mellitus. *Drug Research* 2019;**69**(6):314-22. [PMID: 30103216] |
|  | IMPROVE 2017 | Petrykiv SI, Laverman GD, de Zeeuw D, Heerspink HJ. The albuminuria-lowering response to dapagliflozin is variable and reproducible among individual patients.. *Diabetes, Obesity & Metabolism* 2017;**19**(10):1363-70. [MEDLINE: 28295959] |
|  | Jian 2018 | Jian X, Yang QL, Xiao S, Jing Z, Hu SD. The effects of a sodium- glucose cotransporter 2 inhibitor on diabetic nephropathy and serum oxidized low-density lipoprotein levels. *European Review for Medical & Pharmacological Sciences* 2018;**22**(12):3994-9. [MEDLINE: 29949175] |
|  | Kadowaki 2014 | Kadowaki T, Haneda M, Inagaki N, Terauchi Y, Taniguchi A, Koiwai K, et al. Empagliflozin monotherapy in Japanese patients with type 2 diabetes mellitus: a randomized, 12-week, double-blind, placebo-controlled, phase II trial. *Advances in Therapy* 2014;**31**(6):621-38. [MEDLINE: 24958326] |
|  | Kaku 2014 | Kaku K, Kiyosue A, Inoue S, Ueda N, Tokudome T, Yang J, et al. Efficacy and safety of dapagliflozin monotherapy in Japanese patients with type 2 diabetes inadequately controlled by diet and exercise. *Diabetes, Obesity & Metabolism* 2014;**16**(11):1102-10. [MEDLINE: 24909293] |
|  | Kohan 2014 | Kohan DE, Fioretto P, Tang W, List JF. Long-term study of patients with type 2 diabetes and moderate renal impairment shows that dapagliflozin reduces weight and blood pressure but does not improve glycemic control. *Kidney International* 2014;**85**(4):962-71. [MEDLINE: 24067431] |
|  | LANTERN 2015 | Kashiwagi A, Takahashi H, Ishikawa H, Yoshida S, Kazuta K, Utsuno A, et al. A randomized, double-blind, placebo-controlled study on long-term efficacy and safety of ipragliflozin treatment in patients with type 2 diabetes mellitus and renal impairment: results of the long-term ASP1941 safety evaluation in patients with type 2 diabetes with renal impairment (LANTERN) study. *Diabetes, Obesity & Metabolism* 2015;**17**(2):152-60. [MEDLINE: 25347938] |
|  | Maldonado 2019 | Maldonado M, Cherney D, Frederich R, Liu J, Pong A, Xu J, et al. Effects of ertugliflozin on UACR and eGFR in patients with type 2 diabetes (T2D) and CKD stage 3 [abstract no: SAT-291]. *Kidney International Reports* 2019;**4**(7 Suppl):S130. [EMBASE: 2002179217] |
|  | Mathieu 2015 | Mathieu C, Ranetti AE, Li D, Ekholm E, Cook W, Hirshberg B, et al. Randomized, double-blind, phase 3 trial of triple therapy with dapagliflozin add-on to saxagliptin plus metformin in type 2 diabetes. *Diabetes Care* 2015;**38**(11):2009-17. [PMID: 26246458] |
|  | Nandula 2021 | Nandula SR, Kundu N, Awal HB, Brichacek B, Fakhri M, Aimalla N, et al. Role of canagliflozin on function of CD34+ve endothelial progenitor cells (EPC) in patients with type 2 diabetes. *Cardiovascular Diabetology* 2021;**20**(1):44. [PMID: 33581737] |
|  | NCT01137474 | A study of BMS-512148 (dapagliflozin) in patients with type 2 diabetes and inadequately controlled hypertension on an angiotensin-converting enzyme inhibitor or angiotensin receptor blocker. www.clinicaltrials.gov/ct2/show/results/ NCT01137474. |
|  | Nishimura 2015 | Jinnouchi H, Nozaki K, Watase H, Omiya H, Sakai S, Samukawa Y. Impact of reduced renal function on the glucose- lowering effects of luseogliflozin, a selective SGLT2 inhibitor, assessed by continuous glucose monitoring in Japanese patients with type 2 diabetes mellitus. *Advances in Therapy* 2016;**33**(3):460-79. [MEDLINE: 26846284] |
|  | RED 2020 | van Bommel EJ, Muskiet MH, van Baar MJ, Tonneijck L, Smits MM, Emanuel AL, et al. The renal hemodynamic effects of the SGLT2 inhibitor dapagliflozin are caused by post-glomerular vasodilatation rather than pre-glomerular vasoconstriction in metformin-treated patients with type 2 diabetes in the randomized, double-blind RED trial. *Kidney International* 2020;**97**(1):202-12. [PMID: 31791665] |
|  | Rosenstock 2012 | Rosenstock J, Vico M, Wei L, Salsali A, List JF. Effects of dapagliflozin, an SGLT2 inhibitor, on HbA(1c), body weight, and hypoglycemia risk in patients with type 2 diabetes inadequately controlled on pioglitazone monotherapy. *Diabetes Care* 2012;**35**(7):1473-8. [MEDLINE: 22446170] |
|  | Satirapoj 2019 | Satirapoj B, Korkiatpitak P, Supasyndh O. Effect of sodium- glucose cotransporter 2 inhibitor on proximal tubular function and injury in patients with type 2 diabetes: a randomized controlled trial. *Clinical Kidney Journal* 2019;**13**(3):326-32. [PMID: 31198224] |
|  | SCORED 2020 | Bhatt DL, Szarek M, Pitt B, Cannon CP, Leiter LA, McGuire DK, et al. Sotagliflozin in patients with diabetes and chronic kidney disease. *New England Journal of Medicine* 2021;**384**(2):129-39. [PMID: 33200891] |
|  | Seino 2015 | Seino Y, Inagaki N, Haneda M, Kaku K, Sasaki T, Fukatsu A, et al. Efficacy and safety of luseogliflozin added to various oral antidiabetic drugs in Japanese patients with type 2 diabetes mellitus. *Journal of Diabetes Investigation* 2015;**6**(4):443-53. [MEDLINE: 26221523] |
|  | SOLOIST-WHF 2021 | Bhatt DL, Szarek M, Steg PG, Cannon CP, Leiter LA, McGuire DK, et al. Sotagliflozin in patients with diabetes and recent worsening heart failure. *New England Journal of Medicine* 2021;**384**(2):117-28. [PMID: 33200892] |
|  | Stein 2014 | Stein P, Berg JK, Morrow L, Polidori D, Artis E, Rusch S, et al. Canagliflozin, a sodium glucose co-transporter 2 inhibitor, reduces post-meal glucose excursion in patients with type 2 diabetes by a non-renal mechanism: results of a randomized trial. *Metabolism: Clinical & Experimental* 2014;**63**(10):1296-303. [MEDLINE: 25110280] |
|  | Takashima 2018 | Takashima H, Yoshida Y, Nagura C, Furukawa T, Tei R, Maruyama T, et al. Renoprotective effects of canagliflozin, a sodium glucose cotransporter 2 inhibitor, in type 2 diabetes patients with chronic kidney disease: A randomized open- label prospective trial. *Diabetes & Vascular Disease Research* 2018;**15**(5):469-72. [MEDLINE: 29923427] |
|  | Tanaka 2020 | Tanaka M, Yamakage H, Inoue T, Odori S, Kusakabe T, Shimatsu A, et al. Beneficial effects of ipragliflozin on the renal function and serum uric acid levels in Japanese patients with type 2 diabetes: a randomized, 12-week, open-label, active- controlled trial. *Internal Medicine* 2020;**59**(5):601-9. [PMID: 32115517] |
|  | VERTIS-CV 2020 | Cannon CP, Pratley R, Dagogo-Jack S, Mancuso J, Huyck S, Masiukiewicz U, et al. Cardiovascular outcomes with ertugliflozin in type 2 diabetes. *New England Journal of Medicine* 2020;**383**(15):1425-35. [PMID: 32966714] |
|  | VERTIS RENAL 2018 | runberger G, Camp S, Johnson J, Huyck S, Terra SG, Mancuso JP, et al. Ertugliflozin in patients with stage 3 chronic kidney disease and type 2 diabetes mellitus: the VERTIS RENAL randomized study. *Diabetes Therapy Research, Treatment and Education of Diabetes and Related Disorders* 2018;**9**(1):49-66. [MEDLINE: 29159457] |
|  | Wada 2022 | Wada T, Mori-Anai K, Takahashi A, Matsui T, Inagaki M, Iida M, et al. Effect of canagliflozin on the decline of estimated glomerular filtration rate in chronic kidney disease patients with type 2 diabetes mellitus: a multicenter, randomized, double-blind, placebo-controlled, parallel-group, phase III study in Japan. *Journal of Diabetes Investigation* 2022;**13**(12):1981-9. [MEDLINE: 35861630] |
|  | Weber 2016 | Weber MA, Mansfield TA, Cain VA, Iqbal N, Parikh S, Ptaszynska A. Blood pressure and glycaemic effects of dapagliflozin versus placebo in patients with type 2 diabetes on combination antihypertensive therapy: a randomised, double- blind, placebo-controlled, phase 3 study. *The Lancet Diabetes & Endocrinology* 2016;**4**(3):211-20. [MEDLINE: 26620248] |
|  | Wilding 2012 | Wilding JP, Woo V, Rohwedder K, Sugg J, Parikh S. Dapagliflozin in patients with type 2 diabetes receiving high doses of insulin: efficacy and safety over 2 years. *Diabetes, Obesity & Metabolism* 2014;**16**(2):124-36. [MEDLINE: 23911013] |
|  | Zambrowicz 2015 | Zambrowicz B, Lapuerta P, Strumph P, Banks P, Wilson A, Ogbaa I, et al. LX4211 therapy reduces postprandial glucose levels in patients with type 2 diabetes mellitus and renal impairment despite low urinary glucose excretion. *Clinical Therapeutics* 2015;**37**(1):71-82. [MEDLINE: 25529979] |
